# Pan-Genome Analysis Reveals Evolutionary Dynamics and Functional Divergence of the *NAC* Gene Family in Soybean

**DOI:** 10.3390/plants15132010

**Published:** 2026-06-29

**Authors:** Nan Wu, Yongqi Feng, Xilin Ning, Dan Yao

**Affiliations:** 1 Agronomy College, Jilin Agricultural Science and Technology College, Jilin 132101, China; wunan888@jlnku.edu.cn (N.W.); ningxilin@jlnku.edu.cn (X.N.); 2 Jilin Provincial Seed Management Station, Changchun 130033, China; fyq1990202407@163.com; 3 College of Life Sciences, Jilin Agricultural University, Changchun 130118, China

**Keywords:** core and dispensable genes, genome-wide gene family evolution, NAC, pan-genome, soybean, TE

## Abstract

Soybean (Glycine max) is an important model crop for studying plant functional genes, such as the NAC transcription factor (TF) gene family. The NAC transcription factor (TF) family is one of the largest plant-specific TF families and plays critical roles in plant growth, development, and stress responses. In this study, we performed a pan-genome-wide analysis of *NAC* genes using 29 soybean genomes. A total of 5051 *NAC* genes were identified and clustered into 245 orthologous gene groups (OGGs), including 58 core, 88 soft-core, 32 shell, and 67 cloud groups. Based on phylogenetic relationships, the representative *NAC* OGGs were assigned to 18 subfamilies, 17 of which contained soybean *NAC* genes. Gene duplication analysis indicated that whole-genome duplication (WGD)/segmental duplication was the predominant driver of *NAC* family expansion, accounting for 90.88% of duplication events. Approximately 39.30% of *NAC* genes carried at least one intact transposable element (TE) within 2 kb upstream or downstream regions. *NAC* genes with copy number variation (CNV) harbored more nearby TEs than non-CNV genes (1.54 vs. 1.31 TEs per gene), and dispensable *NAC* genes contained more nearby TEs than core *NAC* genes (1.59 vs. 1.33 TEs per gene). These results indicate a significant association between local TE abundance and *NAC* gene CNV or dispensability. Selection pressure analysis showed that dispensable *NAC* genes had higher Ka, Ks, and Ka/Ks values than core genes, suggesting relatively relaxed evolutionary constraints. Expression profiling across six tissues revealed distinct transcriptional patterns among NAC subfamilies. Structurally conserved subfamilies generally showed broader expression, whereas structurally divergent subfamilies displayed greater expression variability. Regulatory network and Gene Ontology (GO) enrichment analyses suggested that conserved subfamilies were mainly associated with stress responses, while divergent subfamilies were related to cell wall regulation, signal transduction, and ion homeostasis. Further analysis of Wm82 drought RNA-seq data prioritized several putative drought-responsive *NAC* candidates, including Glyma.16G043200, Glyma.06G248900, Glyma.07G050600, Glyma.12G206900, and Glyma.18G261300. Overall, these findings elucidate the mechanisms of expansion and the functional divergence of the *NAC* gene family at the soybean pan-genome level, providing a theoretical basis for understanding *NAC* gene evolution and facilitating future crop improvement.

## 1. Introduction

Soybean (*Glycine max*) is a globally important food and oil crop, rich in protein and oil, and widely used in human food, animal feed, and industrial production. Moreover, soybean exhibits abundant genetic diversity across different ecological environments, making it an important model crop for studying plant functional genes and environmental adaptation mechanisms [1,2]. Transcription factors (TFs) are proteins that regulate downstream gene transcription by specifically recognizing and binding to cis-acting elements [3]. However, previous studies of *NAC* genes in soybean have mainly relied on a single-reference genome, limiting our understanding of intraspecific variation in this family. As central hubs in plant gene expression networks, TFs participate in almost all essential biological processes. They not only coordinate normal growth and development, including cell division, organogenesis, and metabolic pathways, but also serve as key regulators in responses to abiotic stresses (e.g., drought and salinity) and biotic stresses (e.g., pathogen infection) [4].

Among TF families, the *NAC* family (NAM, ATAF1/2, and CUC2) is the fourth largest plant-specific TF family [5,6]. The N-terminus of NAC proteins harbors a highly conserved NAC domain of approximately 150 amino acids, which can be divided into five subdomains (A–E). Subdomains A, C, and D are the most conserved, whereas subdomain A mediates the formation of NAC protein dimers, including both homodimers and heterodimers [7,8]. Subdomains C and D, enriched with positively charged residues, facilitate DNA binding and contain nuclear localization signals (NLSs), thereby ensuring nuclear import and downstream gene regulation [8]. In contrast, subdomains B and E are more variable and can contribute to functional diversification within the *NAC* family [9]. The C-terminal transcriptional regulatory region is highly variable and consists of transcriptional activation or repression modules (TARs) of diverse numbers and lengths, which confer *NAC* transcription factors with considerable functional plasticity [7,9]. Functional investigations have demonstrated that NAC TFs are crucial for numerous developmental processes, including embryogenesis, floral organ differentiation, flowering time regulation, fruit ripening, and secondary cell wall biosynthesis and lignification [8,10]. Increasing evidence also indicates that *NAC* TFs serve as central regulators in plant responses to both biotic stresses (e.g., pathogen infection) and abiotic stresses (e.g., drought and salinity). Furthermore, they are involved in the regulation of multiple phytohormone signaling pathways, such as those mediated by abscisic acid (ABA), jasmonic acid (JA), and salicylic acid (SA) [11,12].

With the rapid progress in high-throughput sequencing technologies, the number of species with reference genomes has increased exponentially over the past two decades, resulting in a surge of studies on gene families [13]. Taking the *NAC* family as an example, the number of identified members varies substantially among species, including 105 in *Arabidopsis*, 75 in rice, 78 in melon, 102 in *Rhododendron delavayi*, 116 in *Thinopyrum elongatum*, 180 in soybean, and 488 in wheat [14,15,16,17,18,19]. However, analyses based on a single-reference genome are insufficient to capture intraspecific genetic diversity, particularly with respect to presence/absence variations (PAVs) and copy number variations (CNVs) [20,21]. The concept of the pan-genome, initially proposed in bacteria, has been widely applied to plants to assess genetic diversity within species [22].

Currently, research on the soybean *NAC* gene family is largely restricted to single cultivars. Within the pan-genome framework, systematic identification and analysis of the *NAC* family are limited. This gap not only constrains a comprehensive understanding of the evolutionary history and conservation of the soybean *NAC* family but also limits deeper insights into the functional diversification of its subfamilies. Therefore, publicly available pan-genomic data from 29 soybean accessions were utilized to systematically identify and analyze the *NAC* gene family, aiming to address the limitations of single-reference genome approaches and to characterize the complete evolutionary landscape of *NAC* genes in soybean. At the pan-genome level, a comprehensive identification of *NAC* family members was conducted, and these genes were classified into core and non-core categories. The origin and expansion mechanisms of the family were further investigated through phylogenetic reconstruction, gene structure and conserved motif analyses, duplication type classification, and evaluation of their association with transposable elements. In addition, the integration of selection pressure analysis, transcriptomic expression profiling, regulatory network construction, and functional enrichment assessments provided systematic insights into evolutionary features of the soybean *NAC* family, which could highlight their potential roles in plant growth, development, and environmental adaptation.

## 2. Results

### 2.1. Identification of NAC Transcription Factors Across 29 Soybean Genomes

**Figure 1:**
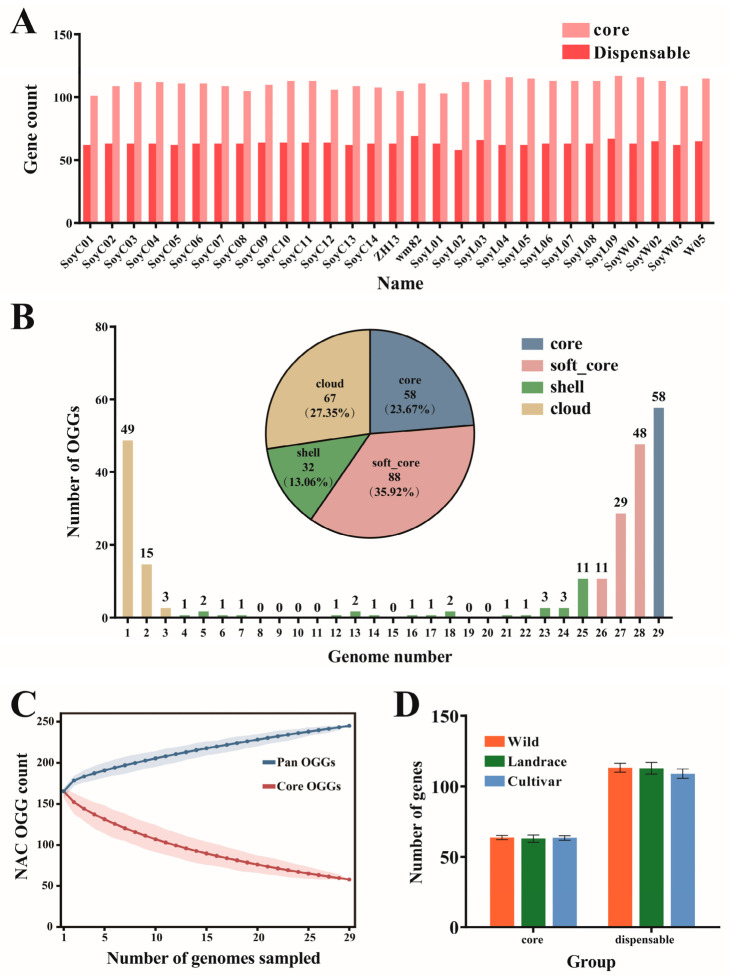
Pan-genome analysis of *NAC* genes based on 29 soybean genomes. (**A**) Comparative distribution of core and dispensable genes across the 29 soybean genomes. Core genes are shown in dark red, and dispensable genes are shown in light red. (**B**) Composition of OGGs at the pan-genome level. The histogram illustrates the frequency distribution of shared OGGs across genomes, and the pie chart displays the relative proportions of core (blue), soft-core (red), shell (green), and cloud (yellow) OGGs. (**C**) Accumulation dynamics of *NAC* OGGs based on 1000 random permutations. The solid line represents the mean OGG number across permutations, and the shaded area represents the 95% confidence interval. (**D**) Comparison of core and dispensable gene counts in wild, landrace, and cultivated soybean genomes.

To investigate the evolutionary characteristics and functions of *GmNAC* genes in soybean, 29 genomes were collected from public databases, including 16 cultivated varieties, nine landraces, and four wild varieties, for genome-wide identification of *NAC* genes. Based on conserved NAC protein domains, a total of 5051 NAC family members were identified. The number of *NAC* genes varied among different accessions, ranging from 163 in cultivar SoyC01 to 184 in landrace SoyL09 (Figure 1A; Supplemental Table S4). According to OrthoFinder classification, 5051 *NAC* genes were clustered into 245 OGGs. This number substantially exceeded the average *NAC* gene count per genome (174), indicating the prevalence of PAV within the soybean NAC family. Among these 245 OGGs, 58 were classified as core and 187 as dispensable, including 88 soft-core, 32 shell, and 67 cloud groups (Figure 1B; Supplemental Table S4). Distribution analysis revealed that the soft-core OGGs contained the largest number of genes (2472; 48.94%), whereas the cloud OGGs were the least represented (89; 1.76%) (Supplemental Table S5).

To assess the accumulation pattern of *NAC* OGGs across the sampled genomes, sequential permutation bootstrap analysis was performed. The mean accumulation curve showed that the number of *NAC* OGGs increased rapidly at early sampling steps and then gradually approached a plateau, suggesting that the 29 genomes captured the major component of the soybean pan-NAC repertoire (Figure 1C; Supplemental Table S6). To further describe *NAC* gene variation among different soybean germplasm groups, the average numbers of core and dispensable *NAC* genes were compared among wild accessions, landraces, and cultivated accessions (Figure 1D). This comparison showed that core *NAC* gene numbers were relatively stable among the three germplasm groups, whereas dispensable *NAC* gene numbers displayed greater variation, with slightly higher average values in wild accessions and landraces than in cultivated accessions.

### 2.2. Pan-Genome-Based Phylogenetic Analysis and Classification of the NAC Gene Family in Soybean

**Figure 2:**
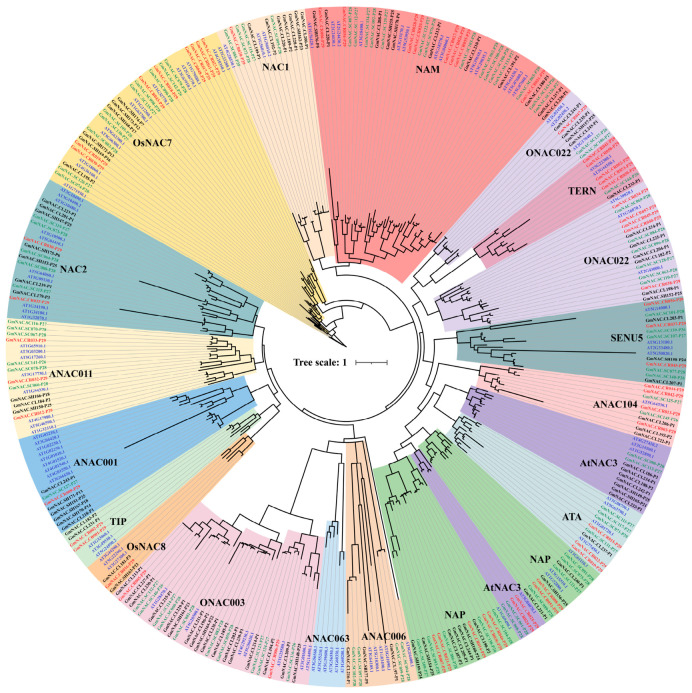
Phylogenetic classification of *NACs* in soybean and *Arabidopsis*. Phylogenetic analysis was performed using *Arabidopsis NACs* (blue) as the classification reference. The tree contained 105 *Arabidopsis NACs* and 245 representative soybean *NAC* genes from OGGs. Soybean *NACs* were classified into three categories based on their conservation across the 29 soybean genomes: core (red), soft-core (green), and shell/cloud (black). The suffix in the soybean *NAC* pan-genome IDs (P1–P29) indicates the number of soybean accessions containing that gene. For example, P29 indicates that this gene is present in all 29 soybean varieties.

A phylogenetic analysis of 245 *NAC* OGGs in soybean was conducted to elucidate the evolutionary dynamics of this gene family. Using the longest protein sequence from each OGG as the representative and referencing the subfamily classification of *Arabidopsis NAC* genes, a phylogenetic tree was constructed. The results demonstrated that all *NAC* genes were categorized into 18 subfamilies (Figure 2; Supplemental Tables S7 and S10), with 13 subfamilies consistent with established *Arabidopsis* classifications. Previously unclassified *Arabidopsis* genes were assigned to five subfamilies, with ANAC001, SENU5, and ANAC063 each gaining one, two, and two additional *Arabidopsis* genes, respectively, compared with earlier classifications. The unclassified *ANAC104* genes were designated as a separate ANAC104 subfamily, whereas the remaining five unclassified *Arabidopsis* genes were placed in the ANAC006 subfamily. Comparative analysis revealed a substantial expansion in 15 soybean *NAC* subfamilies (e.g., *OsNAC7*, *NAC1*, and *NAM*) relative to *Arabidopsis*, whereas ANAC001 and ANAC063 exhibited reduced gene numbers, and only the *OsNAC8* subfamily maintained identical gene counts between the two species. These divergences may reflect functional diversification associated with physiological and ecological adaptations.

The distribution patterns of core and dispensable gene family members may also reflect their evolutionary trajectories. Our analysis demonstrated a non-random distribution of core and dispensable *NAC* genes across subfamilies, with some forming distinct sister clades. Specifically, core *NAC* genes were significantly enriched in conserved subfamilies such as TERN and *OsNAC7*, suggesting that these subfamilies may regulate fundamental physiological processes in soybean, including organ development and stress signal transduction. In contrast, dispensable *NAC* genes were predominantly clustered in subfamilies, such as *AtNAC3*, *ONAC003*, *ANAC001*, and *ANAC006*. This distribution pattern could result from recent gene duplication events and functional diversification driven by species-specific selection pressures (Table 1).

### 2.3. Pan-Genome-Based Analysis of Motif Conservation and Structural Variation in Soybean NAC Transcription Factors

To elucidate the expansion mechanisms and evolutionary relationships of the *NAC* gene family in soybean, comprehensive analyses of protein motifs (Supplementary Figure S1A) and gene structures (Supplementary Figure S1B) were conducted in *NAC* OGGs. The motif analysis revealed three characteristic distribution patterns. The first pattern involved pan-subfamily conserved motifs (e.g., motifs 2, 3, 5, and 6), which likely maintained basal NAC function. The second pattern was comprised subfamily-specific motifs, including motifs 7, 9, 13, and 17 exclusive to ONAC003, motif 12 predominantly in *OsNAC7* (except GmNAC.SH166-P18), motif 19 in ONAC022 (except GmNAC.SH167-P18), and motif 10 in ANAC001 (except GmNAC.SC070-P28). Notably, motif 11 was uniquely present in the dispensable cluster of the *AtNAC3* subfamily, which suggested possible protein module reorganization during evolution. The third pattern involved selectively absent motifs, such as motif 1 missing in ANAC006 and ONAC003 and motif 4 absent in TERN and ONAC003. Compared with core *NACs*, dispensable *NACs* exhibited significantly more frequent motif gains and losses, identifying them as prime candidates for future studies on functional evolution.

In terms of gene structure, the number of exons in soybean *NAC* genes ranged from one to 20 (Supplemental Figure S1B; Supplementary Table S9). A significant positive correlation was observed between exon and intron numbers across *NAC* subfamilies, indicating that the basic splicing structure of *NAC* genes was strictly conserved during differentiation. However, evident divergence was detected among subfamilies: TERN, ATAF, and TIP maintained strict conservation of intron–exon structures, whereas ONAC003, ANAC001, and NAC2 displayed pronounced structural variation. These differences reflect the dynamic balance between functional constraints and adaptive selection pressures experienced by the *NAC* family during soybean evolution.

### 2.4. Analysis of CNV, Gene Duplication, and Transposable Elements in the Soybean NAC Pan-Gene Family

**Figure 3:**
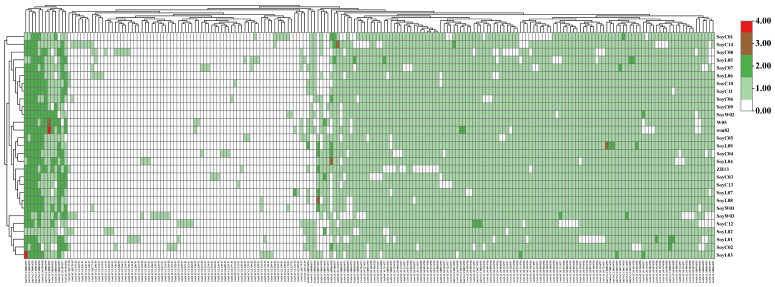
Copy number variation in *NAC* genes across the soybean pan-genome. The heatmap shows CNVs in *NAC* genes across 29 soybean genomes. The color scale represents copy number values ranging from 0 to 4. White indicates gene absence (copy number = 0), and the green-to-red gradient indicates increasing copy number.

To assess CNVs within the *NAC* gene family in the soybean pan-genome, the copy number of each pan-gene was determined (Figure 3; Supplemental Table S11). Overall, the number of genes per pan-gene ranged from one (unique to a single soybean line) to 56 (present as multiple copies in certain genomes). Based on copy number patterns, the 245 *NAC* pan-genes comprised 37 single-copy core genes spanning 11 subfamilies. The remaining 208 pan-genes that exhibited CNVs were categorized into three groups: multi-copy core *NACs*, multi-copy dispensable *NACs*, and single-copy dispensable *NACs*. Among the 21 multi-copy core genes, most (18) were present in two copies in some accessions, whereas *GmNAC.CR009-P29* had three copies, and *GmNAC.CR002-P29* together with *GmNAC.CR007-P29* had four copies. A total of 22 *NAC* genes were identified as dispensable genes, with two to three copies in specific cultivars. In contrast, 165 single-copy dispensable *NAC* genes remained as single copies in the genomes where they were present, suggesting that they may not have undergone recent duplication events.

**Figure 4:**
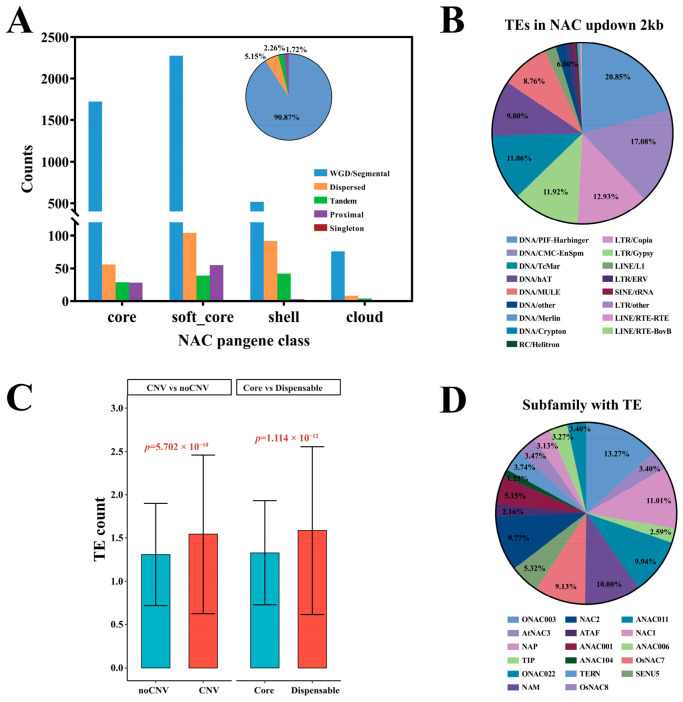
Gene duplication patterns and transposable element distribution of soybean *NAC* genes. (**A**) Distribution of duplication types among core, soft-core, shell, and cloud *NAC* genes. (**B**) Distribution of TE types within the ±2 kb flanking regions of *NAC* genes. (**C**) Number of TEs identified in *NAC* genes with and without CNVs, and in core and dispensable *NAC* genes. *p*-values were calculated using Welch’s two-sample *t*-test and adjusted across the planned comparisons using the Benjamini–Hochberg False discovery rate (FDR) method. (**D**) Distribution of TEs across *NAC* subfamilies.

To evaluate the relationship between conservation level and duplication type, duplication patterns, including WGD/segmental duplication, dispersed duplication, tandem duplication, proximal duplication, and singleton, were quantified across *NAC* genes in different conservation categories (core, soft-core, shell, and cloud) in 29 soybean genomes (Figure 4A; Supplemental Table S12). Among the 5051 *NAC* genes, WGD/segmental duplication was predominant, accounting for 90.88% of duplication events, followed by dispersed duplication (5.15%). Tandem and proximal duplications contributed 2.26% and 1.72%, respectively. No singleton genes (single-copy genes without detected duplication) were detected among the *NAC* family members. WGD/segmental duplication was the most frequent mechanism across all categories, whereas its proportion was higher in core *NACs* than dispensable *NACs*. These findings provide new insights into the molecular mechanisms underlying CNV in *NAC* genes.

Because TE activity is a major driver of structural variation and gene duplication, the TE landscape surrounding *NAC* genes (±2 kb, including gene regions) was characterized (Figure 4B; Supplemental Table S13). Among the 5051 *NAC* genes identified, 1985 (39.30%) contained intact TEs within flanking regions. Notably, 668 genes harbored two to seven intact TEs, suggesting frequent TE insertions. Classification showed that DNA transposons dominated, accounting for 70.80% (2102) of all TEs, with DNA/PIF–Harbinger (20.85%) and DNA/CMC-EnSpm (17.08%) being the most abundant superfamilies. Retrotransposons represented 28.80% primarily long terminal repeats (LTRs), such as Copia and Gypsy (26.40%). For *NAC* genes with CNVs, an average of 1.54 TEs per gene was identified, significantly higher than the 1.31 TEs in *NACs* without CNVs (*p* = 5.702 × 10^−10^) (Figure 4C; Supplementary Figure S2). Dispensable *NACs* contained more TEs on average (1.59) than core *NACs* (1.33) (*p* = 1.114 × 10^−12^). These results indicated a significant correlation between local TE abundance and both CNV and the dispensability of *NAC* genes. The abundance and types of TEs varied significantly across subfamilies (Figure 4D). For instance, ONAC003 (13.27%) and NAP (11.01%) exhibited high TE insertion frequencies, demonstrating enrichment in TE-active genomic regions or frequent insertion events. Conversely, subfamilies, such as ATAF (2.16%) and TIP (2.59%), displayed lower TE proportions and could reflect more stable genomic environments or stronger purifying selection.

### 2.5. Analysis of Natural Selection Pressure on NAC Genes Based on Ka/Ks Estimation

**Figure 5:**
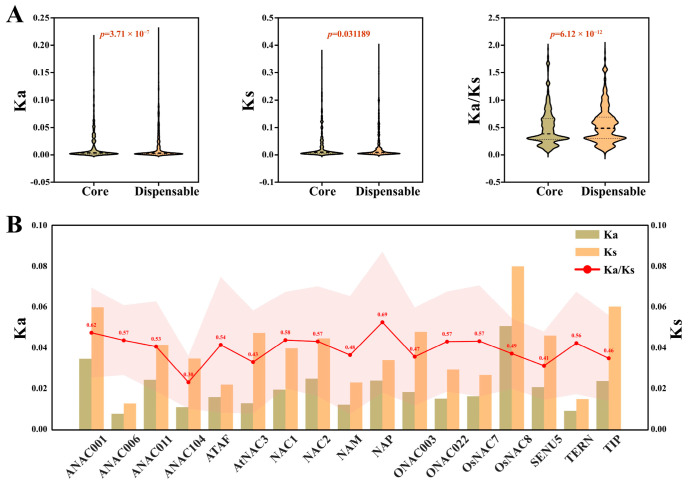
Natural selection analysis of soybean *NAC* transcription factors across the pan-genome. (**A**) Comparison of Ka, Ks, and Ka/Ks values between core and dispensable *NACs*. The black dashed line indicates the mean value. *p*-values were calculated using Welch’s two-sample *t*-test and adjusted using the BH-FDR method. (**B**) Comparison of Ka, Ks, and Ka/Ks values among different *NAC* subfamilies. The average Ka/Ks value is shown above each red dot, and the red shaded area represents the standard deviation of the mean.

To examine the selective pressures acting on soybean *NAC* genes, Ka, Ks, and Ka/Ks values were calculated for each OGG and compared between core and dispensable genes (Figure 5A; Supplemental Table S14). The results indicated that dispensable genes exhibited significantly higher Ka, Ks, and Ka/Ks values than core genes (*p* = 3.71 × 10^−7^, 0.031189, and 6.12 × 10^−12^, respectively). Although the difference in Ks between both groups was statistically significant, the mean values were relatively close, whereas the difference in Ka/Ks was more pronounced, indicating that dispensable genes may experience stronger selective pressures or functional diversification. To assess whether this pattern was affected by filtering of extreme values, we further examined the raw, unfiltered distributions of Ka, Ks, and Ka/Ks values. The raw data showed right-skewed distributions with several extreme values, especially for Ks and Ka/Ks; however, the overall differences between core and dispensable genes were consistent with the filtered results (Supplementary Figure S3; Supplementary Table S15). These results suggest that the observed differences in selective pressure between core and dispensable *NAC* genes are robust and are not solely driven by the filtering procedure.

A systematic analysis of selection pressure on soybean *NAC* genes at the subfamily level was also conducted (Figure 5B). The results indicated that the Ka values were generally low, with most subfamilies ranging between 0.01 and 0.03. In contrast, the Ks values were consistently higher than Ka, mainly distributed within the range of 0.02–0.06. Notably, ANAC001 (0.0598) and TIP (0.0601) exhibited relatively high Ks values, whereas NAM (0.0231) and ATAF (0.0221) had lower values. The discrepancy between Ka and Ks indicated that synonymous substitution rates were generally higher than nonsynonymous substitution rates for most *NAC* genes, consistent with the pattern of strong purifying selection. Further analysis of Ka/Ks ratios revealed that all subfamilies had values below one, suggesting that the evolution of *NAC* genes was predominantly driven by purifying selection. Notably, NAP (0.689) and ANAC001 (0.623) demonstrated relatively higher Ka/Ks ratios, suggesting weaker selective constraints, whereas ANAC104 (0.305) and SENU5 (0.411) exhibited lower ratios, implying a stronger functional conservation. The Ka/Ks values among subfamilies demonstrated significant variations (SD ranging from 0.17 to 0.45), indicating that although *NAC* genes tended to be conserved overall, the intensity of selective pressure differed substantially across lineages. To further evaluate the among-subfamily variation, we also examined the raw, unfiltered distributions of Ka, Ks, and Ka/Ks values across NAC subfamilies (Supplementary Figure S4; Supplementary Table S16). The raw distributions showed that several subfamilies had broader ranges of Ka/Ks values than others, supporting the presence of heterogeneous evolutionary constraints among NAC subfamilies. Such variation may reflect differences in duplication history, functional conservation, or subfamily-specific diversification. In addition, some extreme Ka/Ks values in the raw data may be caused by very small Ks estimates, which can inflate Ka/Ks ratios. Therefore, subfamily-level Ka/Ks patterns were interpreted cautiously, especially for subfamilies with limited numbers of valid pairwise comparisons.

### 2.6. Comprehensive Analysis of Tissue Expression and Interaction Networks in the Soybean NAC Pan-Gene Family

To characterize the transcriptional profiles of *NAC* genes, the expression levels of 245 representative sequences across six tissues (root, stem, leaf, flower, seed, and pod) were analyzed (Supplementary Figure S5; Supplementary Table S17). Relative high expression was defined using the global median threshold of log_2_(TPM + 1) = 1.421, as described in the Methods. Based on their expression patterns, *NAC* sequences were clustered into three major groups (C1–C3). Cluster C1 contained *NAC* genes with high expression across most tissues, C2 included genes highly expressed in zero to two tissues, and C3 consisted of genes exhibiting high expression in three to five tissues. As shown in the figure, no statistically significant difference in expression levels was observed between core and dispensable genes (*p* = 0.643; Supplementary Figure S6A), suggesting that the functional essentiality of a gene is not simply correlated with its expression abundance. Similarly, tissue specificity quantified by the tau index showed no significant difference between core and dispensable *NAC* genes (*p* = 0.310; Supplementary Figure S6B; Supplementary Table S18).

From the perspective of soybean *NAC* subfamilies, significant differences in expression levels across tissues were observed, even within the same subfamily. For example, in the NAC2 subfamily, four representative genes exhibited high expression in all tissues (Cluster C1), eight were highly expressed in most tissues (Cluster C3), and two showed no expression across any tissue (Cluster C2). These results support the view that transcriptional divergence is a common feature in gene family evolution. Furthermore, subfamilies with relatively conserved gene structures, such as ATAF, TIP, TERN, and *AtNAC3*, predominantly contained representative genes with high expression levels (mainly clustered in C1 and C3). In contrast, subfamilies with greater structural variability, such as *ONAC003*, *ANAC001*, and *NAC2*, displayed more heterogeneous expression patterns among their representative genes.

**Figure 6:**
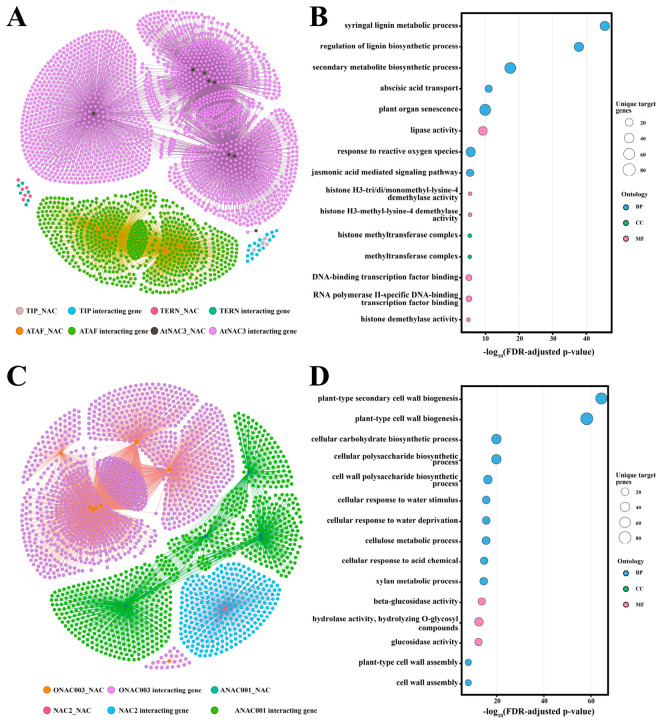
Predicted regulatory networks and GO enrichment of target genes for selected NAC subfamilies. (**A**) Predicted interaction network of *NAC* genes from the TIP, TERN, ATAF, and AtNAC3 subfamilies and their target genes. Different node colors indicate *NAC* genes from each subfamily and their corresponding interacting genes. (**B**) Integrated GO enrichment analysis of target genes from the TIP-, TERN-, ATAF-, and AtNAC3-associated network. (**C**) Predicted interaction network of *NAC* genes from the ONAC003, NAC2, and ANAC001 subfamilies and their target genes. Different node colors indicate *NAC* genes from each subfamily and their corresponding interacting genes. (**D**) Integrated GO enrichment analysis of target genes from the ONAC003-, NAC2-, and ANAC001-associated network. In panels (**B**,**D**), the *x*-axis represents −log10(FDR-adjusted *p*-value), point size indicates the number of unique target genes, and point color indicates GO ontology category, including biological process (BP), cellular component (CC), and molecular function (MF).

To gain further insights into the functional divergence between subfamilies with conserved versus divergent gene structures, a protein–protein interaction network of soybean *NAC* genes was constructed at the Williams 82 (Wm82) cultivar level using the SoyGBN database (Figure 6A,C; Supplementary Tables S19 and S20). Genes from subfamilies with conserved structures and those with divergent structures participated in 5724 and 4127 interaction pairs, respectively. Among these, core, soft-core, and shell *NAC* genes accounted for 2335, 5924, and 1592 interaction pairs, respectively, whereas fewer pairs were identified for cloud *NAC* genes. Notably, the proportion of core *NAC* genes within the structurally conserved group (29.18%) was significantly higher than that within the structurally divergent group (16.11%), indicating that conservation of gene structure may be associated with more fundamental regulatory roles in the gene network. GO enrichment analysis of the predicted target genes further supported functional differences between these two groups (Figure 6B,D). Target genes associated with the structurally conserved subfamilies were enriched in lignin metabolic and biosynthetic processes, secondary metabolite biosynthesis, abscisic acid transport, plant organ senescence, response to reactive oxygen species, and jasmonic acid-mediated signaling. In contrast, target genes associated with the structurally divergent subfamilies were mainly enriched in plant-type cell wall biogenesis, polysaccharide and carbohydrate biosynthetic processes, cellulose and xylan metabolism, and cellular responses to water stimulus and water deprivation. These results suggest that conserved and divergent NAC subfamilies may participate in distinct regulatory modules related to stress responses, secondary metabolism, and cell wall-associated processes.

To further explore the biological significance of these interaction patterns, a functional enrichment analysis was performed on the target genes of each subfamily (Supplementary Figure S7A–D). These results revealed that the target genes of the four structurally conserved *NAC* subfamilies were primarily involved in stress-related functions, although their response pathways varied. The TIP subfamily was associated with intracellular homeostasis, including protein quality control (e.g., ERAD pathway), degradation, and organelle trafficking. The TERN subfamily was characterized by genetic and epigenetic regulation, including the remodeling of large-scale expression programs through chromatin remodeling and histone demethylation. The ATAF subfamily played a central role in stress responses by modulating hormone signaling and autophagy-associated pathways, in addition to defense regulation against pathogen infection, wounding, and leaf senescence. In contrast, the *AtNAC3* subfamily was linked to metabolic reprogramming, including the biosynthesis of lignin and fatty acids as well as the modulation of cell wall composition and metabolites under oxidative stress. Notably, the functional characteristics of TIP-regulated target genes suggest that *TIP* genes may not only align with *Arabidopsis TIP* genes in mediating ER-related signaling but also act as cross-transcriptional regulators of a conserved ER stress response module, spanning signaling, protein turnover, and chromatin remodeling.

Compared with the four structurally conserved *NAC* subfamilies, the non-conserved subfamilies exhibited more diverse functions (Supplementary Figure S7E–G). ONAC003 was primarily enriched in processes related to cell wall biosynthesis and secondary metabolism, suggesting its potential role in structural remodeling and pathogen defense. ANAC001 was associated with both environmental stimulus responses (e.g., drought and light) and cell wall assembly, indicating a bridging role between external signal perception and cell structural adjustment. NAC2 was characterized by the negative regulation of signal transduction and ion transport, particularly involving the ABA pathway and magnesium homeostasis, implying a unique function in stress signaling and ion balance. These results indicated that, in contrast to conserved subfamilies predominantly focused on core stress response pathways, the non-conserved subfamilies evolved diversified functional specializations to adapt to more complex stress environments.

### 2.7. Identification of Drought-Responsive NAC Candidate Genes

**Figure 7:**
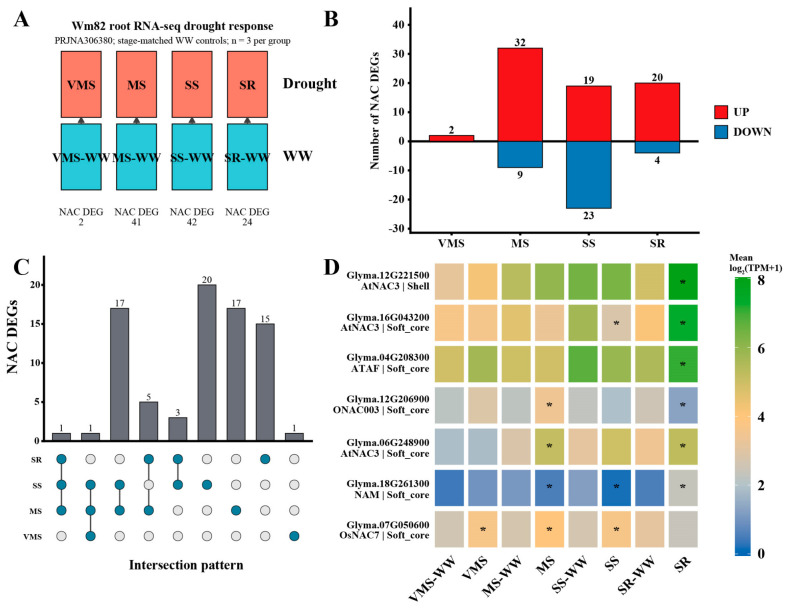
Drought-responsive *NAC* genes identified from Wm82 root RNA-seq data. (**A**) Overview of the Wm82 root RNA-seq dataset under progressive drought treatment. VMS, MS, SS, and SR were compared with their corresponding WW controls, with three biological replicates per group. The number of *NAC* DEGs identified in each comparison is shown below each stage. (**B**) Numbers of upregulated and downregulated *NAC* DEGs in each comparison. Red bars indicate UP genes, and blue bars indicate *DOWN* genes. (**C**) Intersection patterns of *NAC* DEGs across the four comparisons. Filled dots indicate the comparisons included in each intersection, and bars show the number of shared *NAC* DEGs. (**D**) Expression heatmap of selected drought-responsive *NAC* candidate genes across drought-treated and WW samples. Expression values are shown as mean log_2_(TPM + 1). Asterisks indicate significant differential expression relative to the corresponding WW control.

To further identify *NAC* genes potentially involved in drought responses, Wm82 root RNA-seq data under progressive drought treatment were analyzed. Four drought-related stages, including very mild stress (VMS), mild stress (MS), severe stress (SS), and stress recovery (SR), were compared with their corresponding well-watered (WW) controls. A total of two, 41, 42, and 24 *NAC* differentially expressed genes (DEGs) were identified in the VMS, MS, SS, and SR comparisons, respectively (Figure 7A,B). Among them, the MS and SS stages showed the largest numbers of *NAC* DEGs, suggesting stronger transcriptional responses of *NAC* genes during moderate-to-severe drought stress.

Intersection analysis showed that several *NAC* genes responded to multiple drought stages (Figure 7C). Based on repeated drought responsiveness, predicted regulatory network evidence, GO enrichment results, and previously reported drought-related functions, seven *NAC* genes were highlighted as candidate drought-responsive regulators (Figure 7D; Supplementary Table S21). The expression heatmap showed distinct stage-dependent expression patterns among these candidates. For example, Glyma.07G050600 was significantly upregulated at VMS, MS, and SS, whereas Glyma.18G261300 showed significant responses at MS, SS, and SR. In addition, network-supported candidates, including Glyma.06G248900, Glyma.12G206900, Glyma.04G208300, and Glyma.16G043200, showed significant stage-specific responses under drought treatment.

## 3. Discussion

Compared with a single-reference genome, a pan-genome provides a more comprehensive representation of intraspecific genetic diversity, thereby offering a more robust foundation for the systematic identification and functional investigation of gene families [23]. In this study, the total number of OGGs (245) far exceeded the number of *NAC* genes identified in any individual soybean reference genome. Only 23.67% of these OGGs were classified as core genes, a proportion notably lower than the overall core OGG ratio (35.87%) reported for the soybean pan-genome [20]. This indicated that the *NAC* family exhibited greater variability and lower conservation in soybean. Furthermore, the marked reduction in the number of dispensable *NAC* genes in the cultivated varieties was aligned with previously reported losses of genetic diversity during soybean domestication [24].

Regarding duplication modes, the expansion of the soybean *NAC* gene family was primarily driven by WGD/segmental duplication (90.88%), which is highly consistent with the evolutionary history of multiple polyploidization events in soybean [25]. However, duplication patterns varied across *NAC* gene categories, with the core *NACs* being predominantly retained through WGD events, which demonstrated stronger evolutionary constraints on their functions [26]. This conclusion was further supported by the Ka/Ks analysis, which showed that the core genes exhibited significantly lower Ka and Ka/Ks values than dispensable genes, suggesting that they may be subject to long-term purifying selection and tend to maintain functional conservation. In contrast, dispensable *NAC* genes showed more dynamic copy number patterns and higher local TE abundance than core *NAC* genes. Combined with their higher Ka and Ka/Ks values, these genes likely experienced relatively relaxed evolutionary constraints, which may have contributed to functional diversification in certain subfamilies. *NAC* genes with CNVs tend to harbor more nearby TEs, and TE abundance around dispensable *NAC* genes was significantly higher than that around core *NACs*. These patterns suggest that local TE enrichment is associated with *NAC* gene copy number variation and dispensability, although this association should be interpreted as correlative rather than causal because potential confounding factors, such as gene length, GC content, and genomic location, were not explicitly controlled in this study [18,27]. Notably, the significant enrichment of DNA transposons (PIF–Harbinger and CMC-EnSpm) in the genomic neighborhoods of *NAC* genes highlights that they may play important roles in *NAC* expansion and genomic rearrangement [28]. These distinct duplication mechanisms and TE associations not only revealed the complex diversity of the *NAC* gene family in the soybean pan-genome but also suggested that the core *NACs* could maintain fundamental biological functions, whereas dispensable *NACs* may be selectively retained in certain soybean varieties to support adaptive value. Such genes could provide plastic genetic resources for environmental adaptation in soybean [29].

Previous studies have reported that species that undergo extensive genomic duplication events tend to possess larger *NAC* gene families. This not only reflects the asymmetric expansion of this family across different species but also reveals distinct lineage-specific amplification patterns during evolution, ultimately forming species-specific clades [30]. Consistent with Tong et al. [18], who reported the enrichment of dispensable genes in specific subfamilies of *bHLH* genes, dispensable *NAC* genes were also enriched in subfamilies such as *AtNAC3*, *ONAC003*, and *ANAC001*. Within these subfamilies, multiple functional motifs with subfamily-specific distribution were identified. This motif-level specificity suggests that during the expansion of the *NAC* family, asymmetric gene accumulation was accompanied by differential retention and the innovation of functional modules [31]. The expansion and evolution of plant gene families are not affected by a single mechanism but rather result from the combined effects of multiple processes, including WGD, tandem duplication, and TE-mediated duplication [32,33]. Consistent with this view, soybean *NAC* subfamilies displayed distinct evolutionary patterns. For instance, the *AtNAC3* subfamily exhibited relatively lower Ka/Ks values, which may reflect stronger purifying selection. However, enrichment of dispensable *NAC* genes in this subfamily was accompanied by motif 11, which was mainly present in one dispensable *AtNAC3* branch rather than shared by all *AtNAC3* members. Arabidopsis homologs related to this motif 11-containing branch, including ANAC019 and ANAC055, have been implicated in drought-related responses, leaf senescence, reproductive recovery under drought stress, and stress responsive regulatory networks. In contrast, Glyma.16G043200.1/GmNAC12, located in another *AtNAC3* branch lacking motif 11, has been reported to positively regulate drought tolerance in soybean [34]. Since stress-related functions were observed in both *AtNAC3* branches, motif 11 may not determine whether *AtNAC3* members participate in stress responses. Instead, its branch-specific distribution may indicate structural divergence among *AtNAC3* members, which could be associated with more specific regulatory differences. However, this possibility requires further experimental validation [35]. The ONAC003 subfamily exhibited relatively higher Ka/Ks values, and DNA transposons (e.g., PIF–Harbinger and CMC-EnSpm) were significantly enriched near its genes [36]. This pattern suggests that TE abundance is associated with the expansion or structural diversification of this subfamily. The ANAC001 subfamily demonstrated higher Ks values, consistent with the accumulation of mutations over longer evolutionary timescales. Concurrently, TE insertions contribute to the genomic restructuring in this subfamily. This state, which maintains early structural characteristics while incorporating new changes, suggests the potential for functional differentiation. Together, these subfamily-specific patterns may explain the relatively large standard deviations of Ka/Ks values across NAC subfamilies. The broad Ka/Ks distributions suggest non-uniform selection pressure among gene pairs within the same subfamily, possibly reflecting the coexistence of highly conserved members and more divergent paralogs. This evolutionary heterogeneity may provide opportunities for functional diversification, although some extreme Ka/Ks values should be interpreted cautiously because they may be influenced by very small Ks estimates.

The expansion of gene families can be reflected not only in the increase in copy number but also in the functional diversification among their members, a process particularly prominent in plant transcription factor families [37]. In the soybean *NAC* gene family, such diversification can be manifested in different patterns across subfamilies. Previous studies have validated the functions of structurally conserved subfamilies such as TIP, TERN, ATAF, and AtNAC3 in multiple plant species. In *Arabidopsis*, TIP subfamily genes *ANAC062* and *ANAC091* transmit signals from the plasma membrane to the nucleus to directly regulate *UPR* genes [38,39], whereas *Arabidopsis NTL6* induces *PR* gene expression in response to cold stress [40]. Within the TERN subfamily, *Arabidopsis ANAC090* functions as a negative regulator of salicylic acid-mediated leaf senescence [41]. In the ATAF subfamily, soybean *GmNAC035* enhances salt tolerance, and *GmNAC109* participates in auxin signaling to promote lateral root formation and improve abiotic stress resistance [42,43]. In the *AtNAC3* subfamily, soybean *GmNAC12* positively regulates drought tolerance [34], whereas kiwifruit *AcNAC10* increases the resistance to *Pseudomonas syringae* by suppressing jasmonic acid biosynthesis [44]. These studies have demonstrated that structurally conserved NAC subfamilies, such as TIP, TERN, ATAF, and *AtNAC3*, appear to be involved in stress adaptation and developmental regulation across different plant species, which aligns with our interaction network and GO enrichment results in soybean. This suggests their role as core functional modules in the soybean regulatory network. In contrast, structurally divergent subfamilies such as ONAC003, ANAC001, and NAC2 may exhibit greater functional diversification. In the ONAC003 subfamily, rice OsNAC016 interacts with kinases GSK2 and SAPK8 to regulate plant architecture and drought tolerance [45]. In the ANAC001 subfamily, *Arabidopsis ANAC004* enhances Cd tolerance by modulating cell wall fixation, ABA accumulation, and antioxidant capacity [46]. In the NAC2 subfamily, NAC016 promotes drought stress resistance by repressing the expression of the ABA signaling transcription factor *AREB1* [47]. These results are consistent with our enrichment of cell wall biosynthesis, ABA signaling, and related pathways among soybean target genes, although some functions were not detected. Collectively, these findings suggest that structurally divergent *NAC* subfamilies may have retained some conserved functions across species while also potentially acquiring novel functions under different evolutionary contexts, resulting in greater diversity and adaptability.

The integration of pan-genome conservation, predicted regulatory networks, GO enrichment, and Wm82 drought transcriptome data allowed us to prioritize several *NAC* genes as potential drought-responsive candidates. Among these candidates, Glyma.07G050600 and Glyma.18G261300 were significantly differentially expressed in three drought-related comparisons, indicating that they may be involved in sustained or stage-shared drought responses. Glyma.06G248900, Glyma.12G206900, and Glyma.16G043200 were significantly differentially expressed in two comparisons, whereas Glyma.04G208300 and Glyma.12G221500 showed significant responses mainly during the SR stage. These expression patterns suggest that different *NAC* candidates may act at different phases of drought response, including early stress perception, moderate-to-severe drought response, and post-stress recovery. Several of these candidates are supported by previous functional studies. Glyma.16G043200/*GmNAC12* has been reported to positively regulate drought tolerance in soybean [34], and Glyma.06G248900/*GmRD26* responds to PEG, drought, and ABA-related treatments [48]. Glyma.12G221500/*GmNAC004* has been implicated in water stress-associated lateral root development through ABA and auxin-related pathways [49]. In addition, Glyma.07G050600 is associated with pod dehiscence resistance and secondary cell wall thickening [50], while Glyma.04G208300/*GmNAC018* has been reported to enhance salt tolerance [51], suggesting possible roles in cell wall regulation or broader abiotic stress responses. By contrast, Glyma.12G206900/*GmNAC090* and Glyma.18G261300 remain less functionally characterized, and their drought-responsive expression in this study identifies them as underexplored candidates for future validation [15]. Together, these genes provide useful targets for functional studies and molecular design breeding aimed at improving soybean drought tolerance.

Although this study provides a pan-genome-level view of soybean *NAC* genes, several aspects should be considered when interpreting the results. First, the 29 soybean genomes used in this study were generated from different accessions and may differ in assembly quality and annotation completeness, which could affect *NAC* gene identification, copy number estimation, and PAV classification. Second, functional inference was mainly based on sequence homology, conserved motifs, GO enrichment, and predicted regulatory networks; therefore, the proposed functions of candidate *NAC* genes require further experimental validation. Third, the drought transcriptome analysis was performed using Wm82 root RNA-seq data, and these expression patterns may not fully represent responses in other soybean accessions or tissues. This limitation is particularly relevant because the drought-responsive candidates highlighted in Figure 7 were mainly soft-core genes, with one shell gene, indicating that some candidate genes may be absent from certain accessions. Therefore, their drought-responsive roles and breeding potential may depend on accession-specific genetic backgrounds and should be further evaluated across diverse soybean germplasms. Finally, TE annotations were computationally predicted and may include false positives or false negatives, which could influence the inferred association between TE abundance, CNV, and *NAC* gene diversification. Future studies integrating high-quality accession-specific genome assemblies, multi-accession transcriptomes, and experimental validation such as overexpression, knockout, or gene-editing assays will be necessary to confirm the biological roles and breeding value of these candidate *NAC* genes.

## 4. Materials and Methods

### 4.1. Data Sources for the Soybean Pan-Genome and Identification of NAC Genes

Genomic sequences and annotation files (GFF3 format) of 29 soybean accessions were obtained from the SoyMD database (https://yanglab.hzau.edu.cn/SoyMD/#/, accessed on 25 July 2025) [52]. These accessions were selected because they correspond to the 29 soybean genomes used in the soybean pan-genome study by Liu et al. [20], including cultivated accessions, landraces, and wild soybean accessions. Detailed information for the 29 genomes, including accession name, germplasm type, data source, and accession/BioProject information, is provided in Supplementary Table S1. A total of 105 Arabidopsis NAC protein sequences were retrieved from The Arabidopsis Information Resource (TAIR, https://www.arabidopsis.org/, accessed on 25 July 2025) and utilized as the queries for local BLAST (https://www.nlm.nih.gov/ncbi/workshops/2023-08_BLAST_evol/databases.html, accessed on 20 April 2026) searches against the soybean pan-genome. Furthermore, domain scanning was performed with HMMER (HMMER 3.3.2 version; http://hmmer.org/) by applying the NAC domain (PF02365) downloaded from the Pfam database (http://ftp.ebi.ac.uk/pub/databases/Pfam/releases/Pfam35.0/, accessed on 25 July 2025), with an E-value cutoff of 1 × 10^−5^ applied to both methods. Only sequences that satisfied both BLAST (v2.16.0+) and HMMER (v3.4) criteria while containing the conserved NAC domain were retained for subsequent analyses.

### 4.2. Classification of Core and Dispensable NAC Genes in the Soybean Pan-Genome

OrthoFinder (v3.1.0) was employed to analyze the orthologous groups (OGGs) of *NAC* gene family members across 29 soybean genomes [53]. Sequence homology detection in OrthoFinder was performed using BLAST, followed by orthologous group clustering using the Markov Cluster Algorithm (MCL) with the inflation parameter set to two. Phylogenetic reconstruction of gene trees was conducted using Multiple Sequence Alignment (MSA)-based methods. Following the PAV-based classification strategy used by Tong et al. [16], OGGs were categorized according to their presence/absence patterns across the 29 soybean genomes. Specifically, OGGs were classified into four categories: core genes (present in all 29 varieties, 100%), soft-core genes (present in 26–28 varieties ≥90%), shell genes (present in four to 25 varieties, >10%), and cloud genes (present in only one to three varieties, ≤10%). To facilitate downstream analysis, the longest protein sequence from each OGG was selected as the representative sequence for that gene cluster.

### 4.3. Phylogenetic Tree Construction

Multiple sequence alignment of NAC protein sequences was conducted using MAFFT (v7.505) (maxiterate = 1000) [54]. An unrooted maximum likelihood phylogenetic tree was constructed using IQ-TREE (version 1.6.12) by applying the optimal JTT + R9 substitution model determined by ModelFinder [55]. Branch support values were calculated from 1000 bootstrap replicates using SH-aLRT testing. Final tree visualization and annotation were performed using iTOL (http://itol.embl.de/, accessed on 25 August 2025) [56].

### 4.4. Analysis of Conserved Motifs and Gene Structures

Conserved motifs were identified using MEME (version 5.5.8) (https://meme-suite.org/meme/, accessed on 6 August 2025) with the maximum number set to 20, while other parameters were kept at default values [57]. The GFF3 annotation files of 29 soybean accessions were merged into a unified file. Subsequently, TBtools (v2.356)was employed to integrate and visualize gene structure annotations and conserved motif distributions [58].

### 4.5. Gene Duplication Type and Transposable Element (TE) Identification

Protein sequences from 29 soybean accessions were subjected to all-against-all BLASTP (v2.16.0+) analysis with an E-value cutoff of 1 × 10^−5^. For each query, only the top five BLASTP hits were retained, according to the software specifications. The duplication types of *NAC* genes in each variety were then classified using the duplicate_gene_classifier program in MCScanX (v1.0.0) [59].

TEs were annotated in the whole-genome sequences of 29 soybean accessions using HiTE (v3.3.3) with default parameters [60]. The identified TEs were subsequently analyzed for co-localization with *NAC* gene regions (including the gene body and 2000 bp upstream and downstream flanking regions) using BEDTools (v2.31.1) [61].

### 4.6. Analysis of Selection Pressure on the NAC Gene Family

Pairwise Ka, Ks, and Ka/Ks values were calculated after generating homologous sequence alignments using ParaAT (v2.0) [62]. The calculations were performed using KaKs-Calculator 2.0 with the Yang–Nielsen (YN) method [63]. For OGGs containing multiple *NAC* gene copies, pairwise comparisons were performed among homologous gene copies within the same OGG, and valid pairwise results were retained for downstream analysis. Pairwise results with missing or invalid Ka, Ks, or Ka/Ks values were excluded. To reduce the influence of extreme outliers, only values within the range of zero to two were retained in the filtered dataset used for the main statistical comparisons and visualization. The raw, unfiltered Ka, Ks, and Ka/Ks distributions were retained and examined as a robustness check.

### 4.7. Expression Analysis of Soybean NAC Genes

The expression levels (TPM) of 245 representative genes across six tissues (root, stem, leaf, flower, seed, and pod) were obtained from the SoyMD database (accessed on 20 August 2025, Supplementary Table S2). The data were log_2_-transformed [log_2_(TPM + 1)] and subjected to clustering analysis using the HeatMap module in TBtools. A relative high-expression threshold was defined as the global median of log_2_(TPM + 1) values across all representative NAC OGGs and tissues. Genes with log_2_(TPM + 1) values greater than or equal to this threshold were considered relatively highly expressed in the corresponding tissue. Tissue specificity was quantified using the tau index. For each gene, tau was calculated as τ = Σ(1 − xi)/(n − 1), where xi represents the expression level in tissue i normalized by the maximum expression level of that gene across all tissues, and n represents the number of tissues analyzed. Tau values range from zero to one, with values closer to one indicating stronger tissue-specific expression and values closer to zero indicating broader expression across tissues [64,65].

To further identify drought-responsive *NAC* genes, an independent RNA-seq dataset of soybean cultivar Wm82 was downloaded from the NCBI SRA database (accession ID: PRJNA306380). This dataset included root samples under VMS, MS, SS, and SR, each with a corresponding well-watered control and three biological replicates (Supplementary Table S3). Raw sequencing reads were quality controlled and trimmed using fastp (v0.23.4) [66]. Clean reads were aligned to the Wm82 reference genome using HISAT2 (v2.2.1) [67], and gene-level read counts were generated using featureCounts (v2.0.6) [68]. Differential expression analysis was performed using DESeq2 (v1.42.0) in R (v4.5.0) [69]. Four pairwise comparisons were conducted: VMS vs. VMS-WW, MS vs. MS-WW, SS vs. SS-WW, and SR vs. SR-WW. Genes with an adjusted *p*-value (padj) < 0.05 and |log_2_ fold change| ≥ 1 were considered DEGs.

### 4.8. Interaction Network Construction and GO Enrichment Analysis

The soybean gene regulatory network was downloaded from the SoyTFBase database (http://www.soytfbase.cn, accessed on 18 August 2025) [70]. To reduce false positives, a two-step filtering strategy was applied. First, gene pairs were retained if any of the AtDAP (Arabidopsis DAP-seq-based TF binding occupancy), SoyDAP (Soybean DAP-seq-based TF binding occupancy), or OCR (open chromatin region-based chromatin accessibility) indicators were positive. Second, gene pairs with all three negative indicators were retained only if at least one of PCC (Pearson correlation coefficient), GENIE3 (ensemble-of-trees-based gene network inference), cCOE (evolutionarily conserved co-expression), or PWM (position weight matrix-based promoter motif evidence) values exceeded 0.9. Gene pairs that met either condition were considered high-confidence interaction candidates. The interaction network was visualized using Gephi (v0.10.0) with the Fruchterman–Reingold force-directed layout algorithm under default parameter settings [71]. The GO enrichment results of target genes from corresponding subfamilies were obtained from the SoyMD database and visualized using R (v4.5.0) with the ggplot2 (v3.5.1) package.

### 4.9. Statistical Analysis

Statistical analyses were performed using R (v4.5.0) [72]. For two-group comparisons in Figure 4A,C, data distributions were inspected using violin/box plots and Q-Q plots. Homogeneity of variance was evaluated using the median-centered Levene test (Brown–Forsythe test) [73]. Because equal variance could not be assumed for these comparisons, Welch’s two-sample *t*-test was used as the primary test to compare group means [74]. For skewed or count-based variables, Wilcoxon rank-sum tests were additionally performed as non-parametric robustness checks [75]. For multiple planned comparisons within the same figure panel, *p*-values were adjusted using the Benjamini–Hochberg FDR method [76]. Specifically, *p*-values from the two planned comparisons in Figure 4C and from the three Ka, Ks, and Ka/Ks comparisons in Figure 4A were adjusted separately. Statistical significance was defined as FDR-adjusted *p* < 0.05. Diagnostic plots used for distribution assessment and normality evaluation were generated using the ggplot2 package in R (v4.5.0) [77], whereas other figure visualizations were generated using GraphPad Prism 9 and matplotlib (v3.9.0) in Python (v3.12.1) [78].

## 5. Conclusions

Pan-genome analysis of the *NAC* gene family identified 5051 *NAC* genes in soybean. These genes were classified into 245 OGGs, substantially exceeding the 180 reported in the Wm82 reference genome. Among them, 245 OGGs were further categorized into 58 core, 88 soft-core, 32 shell, and 67 cloud groups. The reduction in dispensable *NAC* genes in cultivated varieties suggested a potential loss of genetic diversity during soybean domestication. Analyses of gene duplication, TE distribution, and selective pressures revealed that core *NACs* were primarily derived from WGD/segmental duplication, whereas dispensable *NACs* showed higher local TE abundance and more dynamic evolutionary patterns. Across different subfamilies, *NACs* appeared to evolve through multiple mechanisms, including whole-genome duplication, tandem duplication, and transposon-mediated events. Transcriptome analysis of 245 representative genes revealed no simple correlation between functional essentiality and expression levels. Structurally conserved subfamilies exhibited high expression across multiple tissues, whereas structurally divergent subfamilies displayed more variable expression patterns. The integration of predicted interaction networks and GO enrichment further suggested that conserved subfamilies may be mainly associated with core stress responses, whereas divergent subfamilies may contribute to broader functional diversification and adaptive potential. By integrating pan-genome variation, predicted regulatory networks, GO enrichment, and Wm82 drought transcriptome data, several putative drought-responsive NAC candidates were prioritized, including Glyma.16G043200, Glyma.06G248900, Glyma.07G050600, Glyma.12G221500, Glyma.04G208300, Glyma.12G206900, and Glyma.18G261300. In conclusion, compared with traditional reference genome-based studies, pan-genome family analysis presents a more comprehensive depiction of gene evolutionary dynamics within a species. This approach not only offers new insights into the functional evolution of soybean *NAC* genes but also provides candidate gene resources and theoretical support for future functional validation and molecular design breeding aimed at improving soybean stress tolerance.

## Figures and Tables

**Table 1:** Distribution of *NACs* in *Arabidopsis* and soybean and functionally characterized *NACs* based on the literature review.

Subfamily	Arab.	Soyb.	CoreSingle	withCNV	Characterized Genes and Ref	Representative Function
OsNAC7	13	24	4	20	AT1G32770.1/AtANAC012 [S1];BpNAC012 [S2];AT1G71930.1/VND7 [S3];AT4G10350.1/ANAC070 [S12]	Negatively regulates xylem fiber development in Arabidopsis. Modulates abiotic stress responses and secondary wall biosynthesis. Regulates aluminum tolerance by controlling cell wall xyloglucan content and its aluminum-binding capacity.
NAC1	3	12	0	12	MaNAC5 [S19];AT4G28530.1/ANAC074 [S23]	Induces pathogen resistance ANAC074 binds to the promoters of ethylene-responsive and stress responsive genes via NRS1, NRS2, or MybSt1 cis-elements.
NAM	13	34	7	27	Glyma.05G025500 [S7];ClNAC100 [S11];AT3G04060.1/ANAC046 [S25];AT5G18270.1/ANAC087 [S25]	Enhances resistance to Phytophthora sojae. Regulates lignin biosynthesis. Modulates watermelon plant height and fruit size. Controls the rate of endosperm degradation and cellular debris clearance.
NAC2	10	14	2	12	AT5G04410.1/ANAC078 [S26];AT1G34180.1/ANAC016 [S39]	Inhibits auxin biosynthesis. Inhibition of AREB1 transcription to promote drought stress response.
ANAC011	8	12	2	10	AT4G17980.1/ANAC071 [S22];AT5G46590.1/ANAC096 [S22]	Participates in the “cambialization” process.
TIP	3	4	0	4	AT5G24590.2/ANAC091 [S27];AT3G49530.1/ANAC062 [S28]	The endoplasmic reticulum stress signal is transmitted from the plasma membrane to the nucleus.
OsNAC8	3	3	0	3	AT2G27300.1/ANAC040 [S24];AT3G44290.1/ANAC060 [S24]	Inhibits seed dormancy in Arabidopsis.
ONAC003	7	29	0	29	MaNAC1 [S20];AT3G01600.1/ANAC044 [S29];OsNAC016 [S38]	Involved in banana fruit ripening. Phosphorus remobilization. Regulating plant structure and drought tolerance.
ANAC001	9	8	0	8	AT1G02230.1/ANAC004 [S30]	Cd tolerance.
ONAC022	6	24	6	18	Glyma.02G284300 [S8];MaNAC [S20]2;LjNAC094 [S31];AT2G17040.1/ANAC036 [S32]	Enhances glycinin bioproduction. Bolsters pathogen defense. Regulates banana fruit ripening. Modulates nitrate-induced nodule senescence. Promotes leaf cell growth.
TERN	2	7	5	2	AT5G22380.1/ANAC090 [S33]	Accelerates leaf senescence.
NAP	4	26	3	23	Glyma.01G051300 [S5];SlNAP1 [S14];AcNAC1 [S15];AcNAC2 [S15];SsNAC106 [S16];OsNAC103 [S17];ZmNAC89 [S18]	Enhances root development and significantly increases antioxidant enzyme activity, improving drought resistance. Regulates gibberellin-dependent fruit ripening. Activates xyloglucan endotransglucosylase/hydrolase (XTH) genes to control kiwifruit softening during maturation. Modulates fruit development. OsNAC103 negatively regulates rice plant height by influencing phytohormone-mediated cell cycle and crosstalk. Participates in maize salt tolerance.
ATAF	4	8	1	7	Glyma.06G114000 [S4];Glyma.14G152700 [S9];AT1G01720.1/ATAF1 [S13]	Reduces oxidative damage and fine-tunes salt-responsive genes. Enhances lateral root formation and abiotic stress tolerance. Promotes growth under fluctuating light conditions.
AtNAC3	4	13	2	11	Glyma.16G043200 [S6];CmNAC34 [S10];AcNAC10 [S21];AT1G52890.1/ANAC019 [S34];AT3G15500.1/ANAC055 [S35]	Positively regulates soybean drought tolerance. Positively regulates fruit ripening. Enhances kiwifruit resistance to Pseudomonas syringae. Drought response and floral development. Leaf stress response and senescence.
ANAC063	6	0	0	0	AT3G55210.1/ANAC063	-
ANAC104	1	9	2	7	PopNAC128 [S37]	Functions in the development of lignified fiber cells and rays.
SENU5	4	11	3	8	BrNAC041 [S36]	Participates in ABA-GA crosstalk to regulate leaf senescence in Brassica rapa var.
ANAC006	5	7	0	7	AT1G03490.1/ANAC006	-
Total	105	245	37	208	-	-

Note: Subfamily classification was performed according to the phylogenetic analysis shown in Figure 2. For each subfamily, the numbers of *NAC* genes in Arabidopsis and the soybean pan-genome were calculated. Well-characterized *NAC* genes and their reported functions were compiled from published studies. The reference labels S1–S39 indicate supplementary references supporting the characterized gene information; full bibliographic details are provided in Supplementary Table S8.

## Data Availability

Data supporting the findings of this study are included in the manuscript, methods, and Supplementary Files.

## Supplementary Materials

The following supporting information can be downloaded at: https://www.mdpi.com/article/10.3390/plants15132010/s1.

## Abbreviations

The following abbreviations are used in this manuscript:

ABA	Abscisic acid
AtDAP	Arabidopsis DAP-seq-based TF binding occupancy
BLAST	Basic Local Alignment Search Tool
BLASTP	Protein–protein BLAST
BP	Biological process
CC	Cellular component
cCOE	Evolutionarily conserved co-expression
CMC-EnSpm	CACTA-like transposon superfamily
CNV	Copy number variation
DAP-seq	DNA affinity purification sequencing
DEG	Differentially expressed gene
DNA	Deoxyribonucleic acid
ER	Endoplasmic reticulum
ERAD	Endoplasmic-reticulum-associated degradation
FASTQ	Text-based format for sequencing reads and quality scores
FDR	False discovery rate
GENIE3	Gene network inference with ensemble of trees
GFF3	General Feature Format version 3
GO	Gene Ontology
JA	Jasmonic acid
Ka	Nonsynonymous substitution rate
Ka/Ks	Ratio of nonsynonymous to synonymous substitution rates
Ks	Synonymous substitution rate
LTR	Long terminal repeat
MAFFT	Multiple Alignment using Fast Fourier Transform
MCL	Markov Cluster Algorithm
MCScanX	Multiple Collinearity Scan toolkit X
MEME	Multiple EM for Motif Elicitation
MF	Molecular function
MS	Mild stress
MSA	Multiple sequence alignment
NAC	NAM, ATAF1/2, and CUC2
NLS	Nuclear localization signal
OCR	Open chromatin region
OGG	Orthologous gene group
PAV	Presence/absence variation
PCC	Pearson correlation coefficient
PCR	Polymerase chain reaction
PIF–Harbinger	PIF/Harbinger DNA transposon superfamily
PR	Pathogenesis-related
PWM	Position weight matrix
qRT-PCR	Quantitative reverse-transcription PCR
RNA	Ribonucleic acid
RNA-seq	RNA sequencing
SA	Salicylic acid
SD	Standard deviation
SH-aLRT	Shimodaira–Hasegawa approximate likelihood ratio test
SoyDAP	Soybean DAP-seq-based TF binding occupancy
SoyGBN	Soybean gene regulatory network database
SoyMD	Soybean multi-omics database
SoyTFBase	Soybean transcription factor database
SR	Stress recovery
SS	Severe stress
TAIR	The Arabidopsis Information Resource
TAR	Transcriptional activation or repression region
TE	Transposable element
TF	Transcription factor
TPM	Transcripts per million
UPR	Unfolded protein response
VMS	Very mild stress
WGD	Whole-genome duplication
Wm82	Williams 82
WW	Well-watered
YN	Yang–Nielsen method
