# Supplementary figures and images for: Pan-Genome Analysis Reveals Evolutionary Dynamics and Functional Divergence of the NAC Gene Family in Soybean
Source: Plants (Basel). 2026 Jun 29;15(13):2010. doi: 10.3390/plants15132010 (PMC13364366; doi:10.3390/plants15132010)

## A

## Motif Pattern

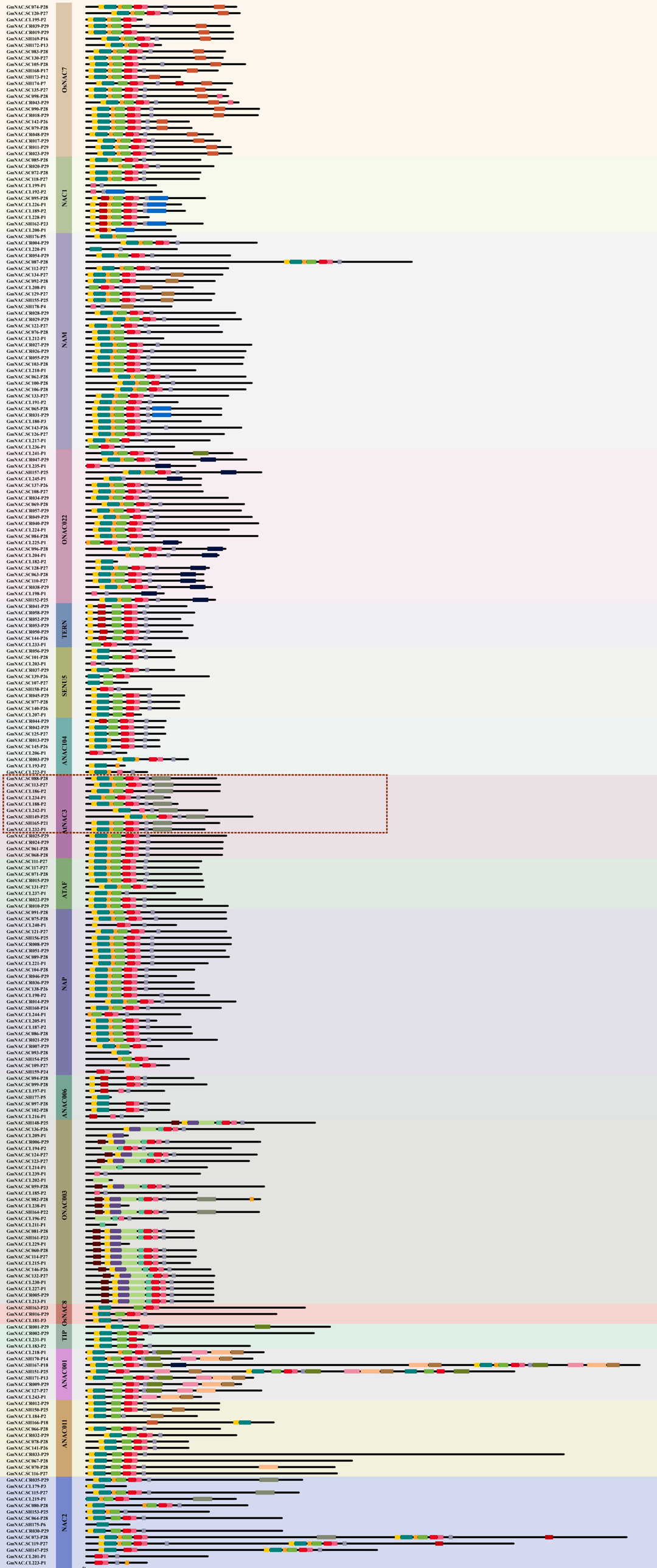

## B.

## Gene structure

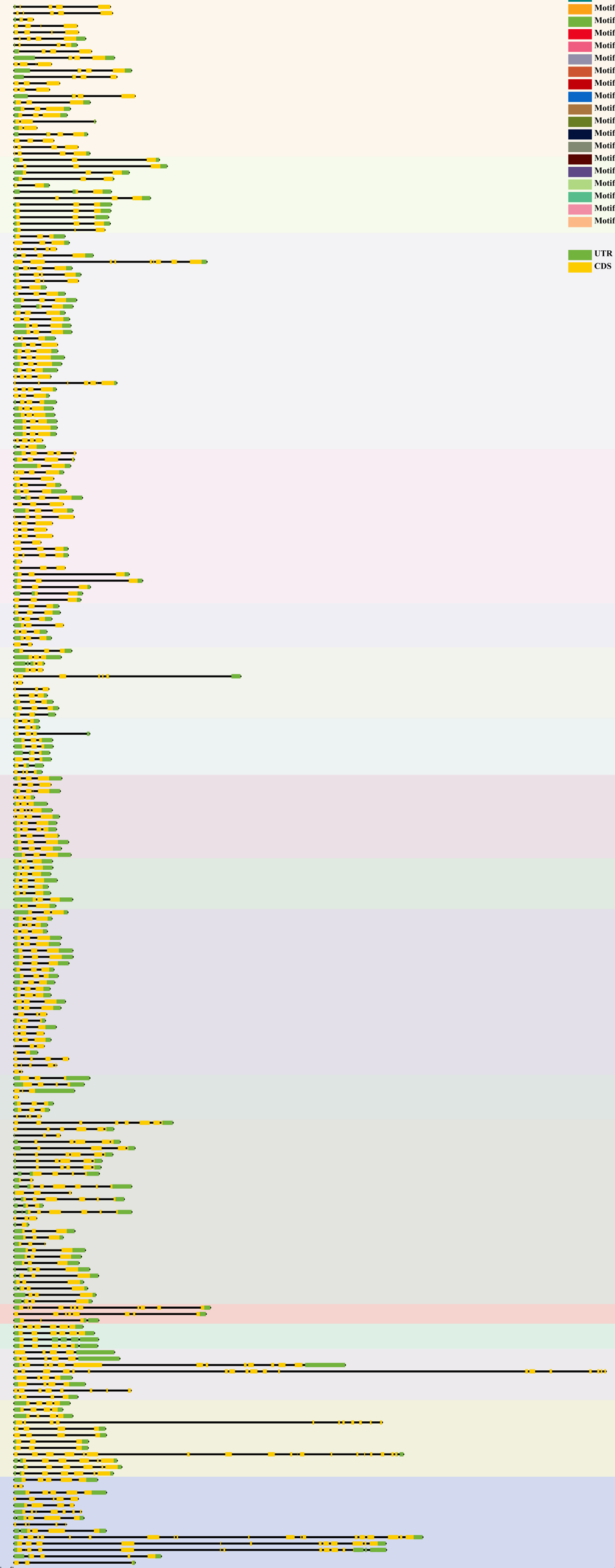

Supplement: Supplementary file 1 [file plants-15-02010-s001.zip › plants-4303159-supplementary figures/Supplementary Figures/Supplementary Figure S1.pdf]

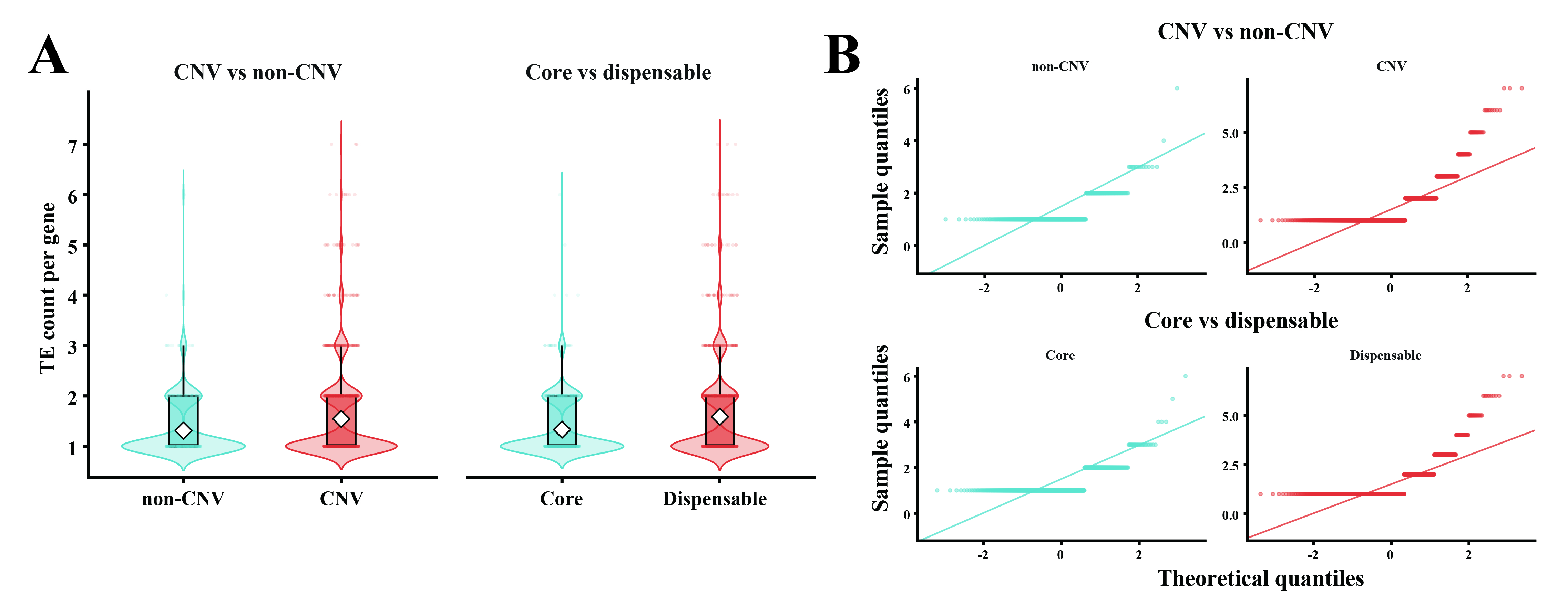

Supplement: Supplementary file 1 [file plants-15-02010-s001.zip › plants-4303159-supplementary figures/Supplementary Figures/Supplementary Figure S2.jpg]

**A****CNV vs non-CNV**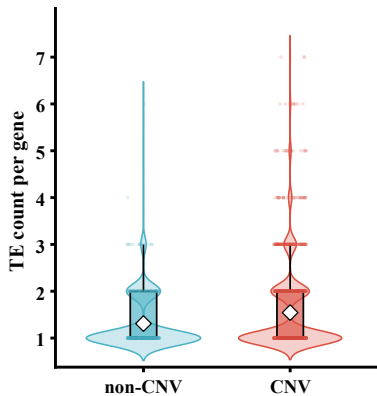**Core vs dispensable**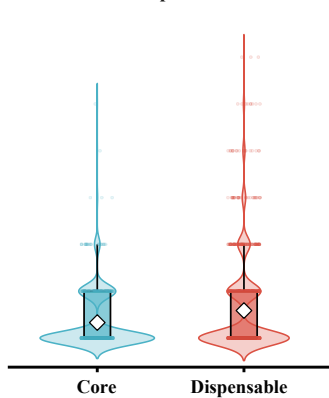**B****CNV vs non-CNV**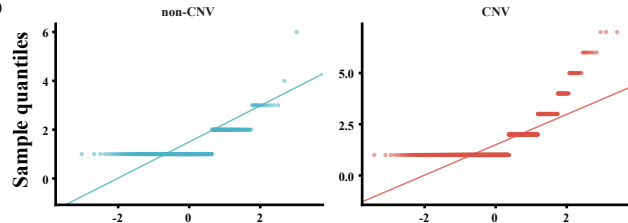**Core vs dispensable**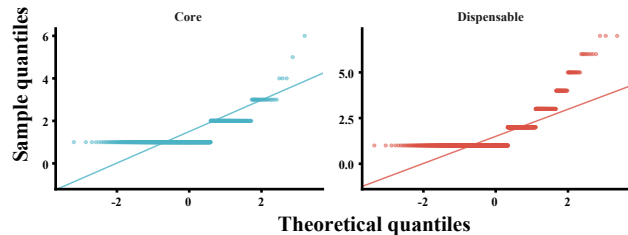

Supplement: Supplementary file 1 [file plants-15-02010-s001.zip › plants-4303159-supplementary figures/Supplementary Figures/Supplementary Figure S2.pdf]

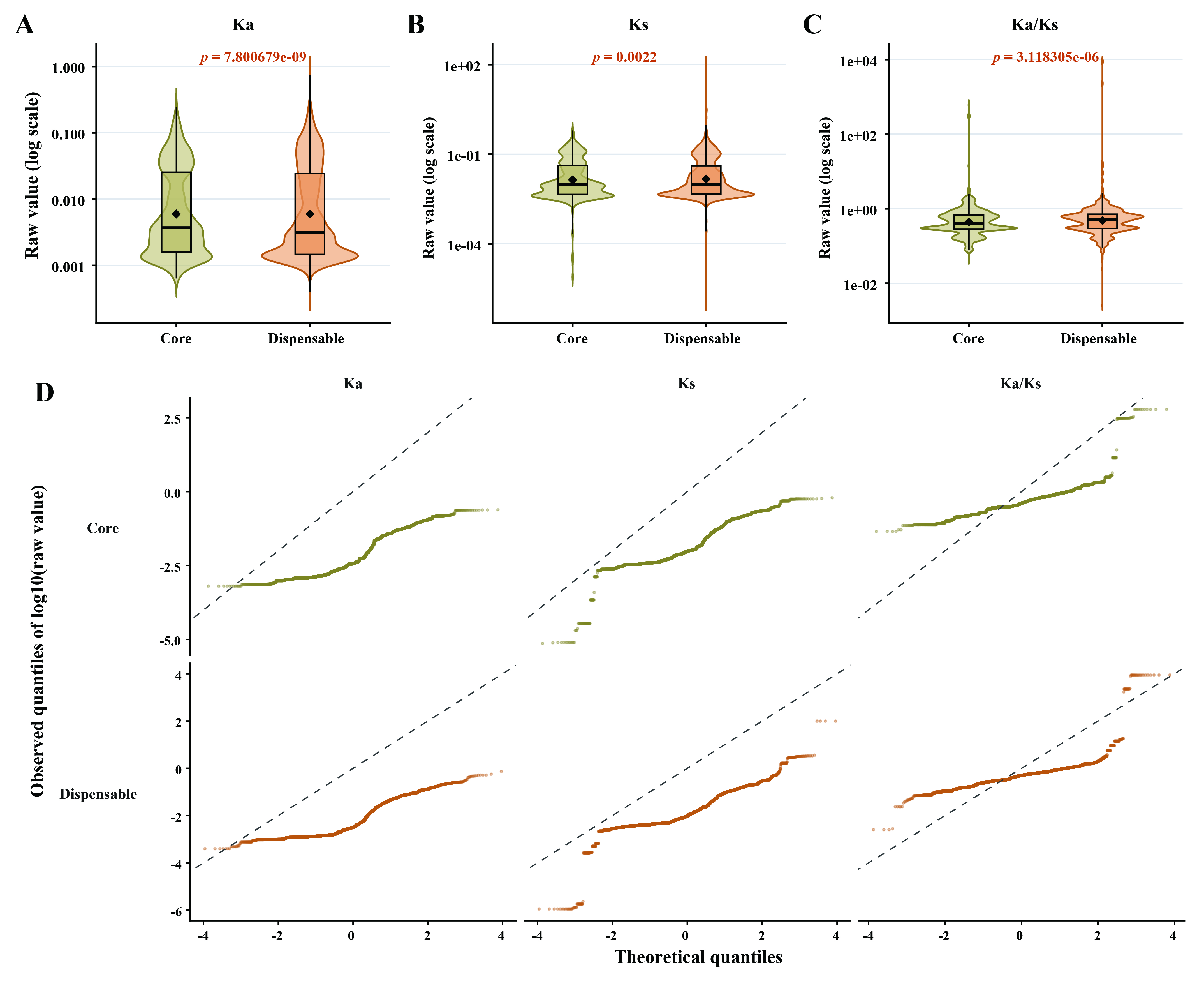

Supplement: Supplementary file 1 [file plants-15-02010-s001.zip › plants-4303159-supplementary figures/Supplementary Figures/Supplementary Figure S3.jpg]

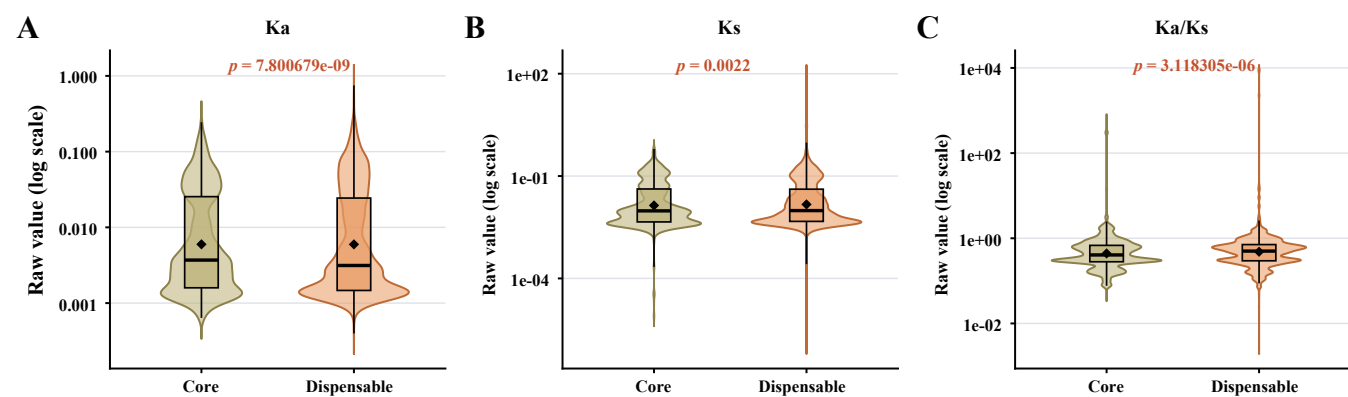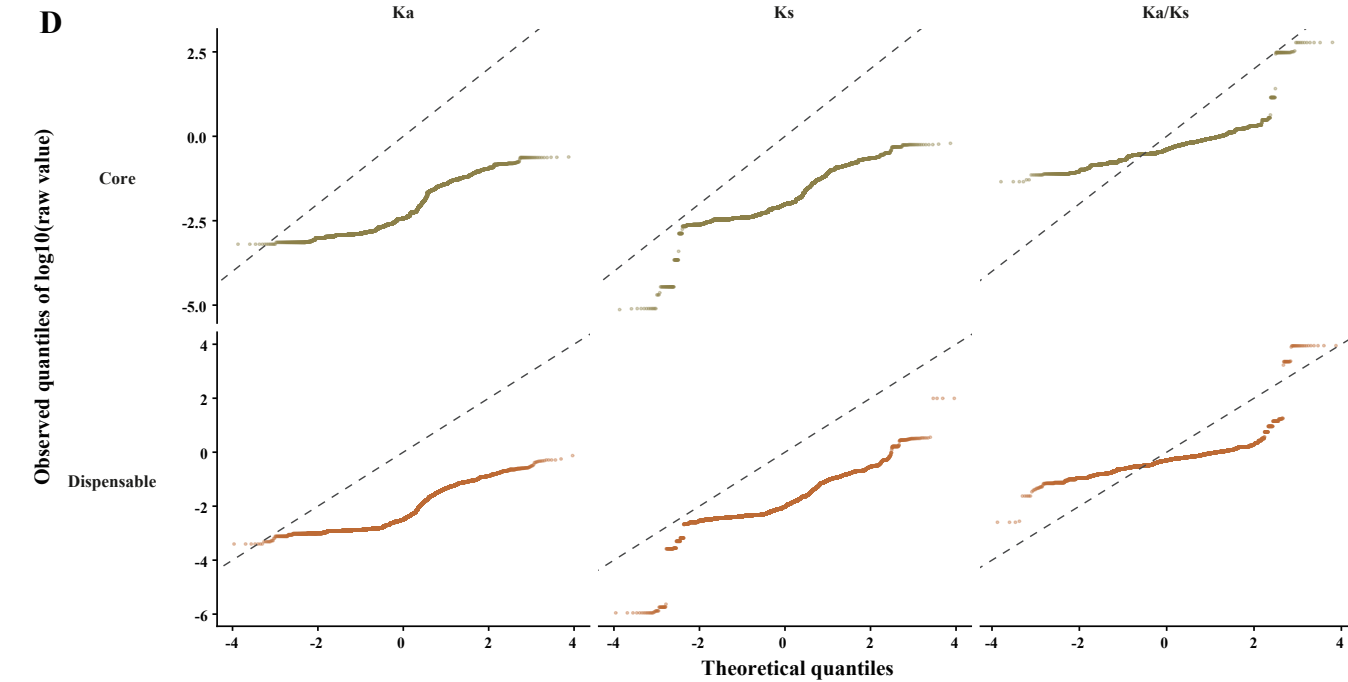

Supplement: Supplementary file 1 [file plants-15-02010-s001.zip › plants-4303159-supplementary figures/Supplementary Figures/Supplementary Figure S3.pdf]

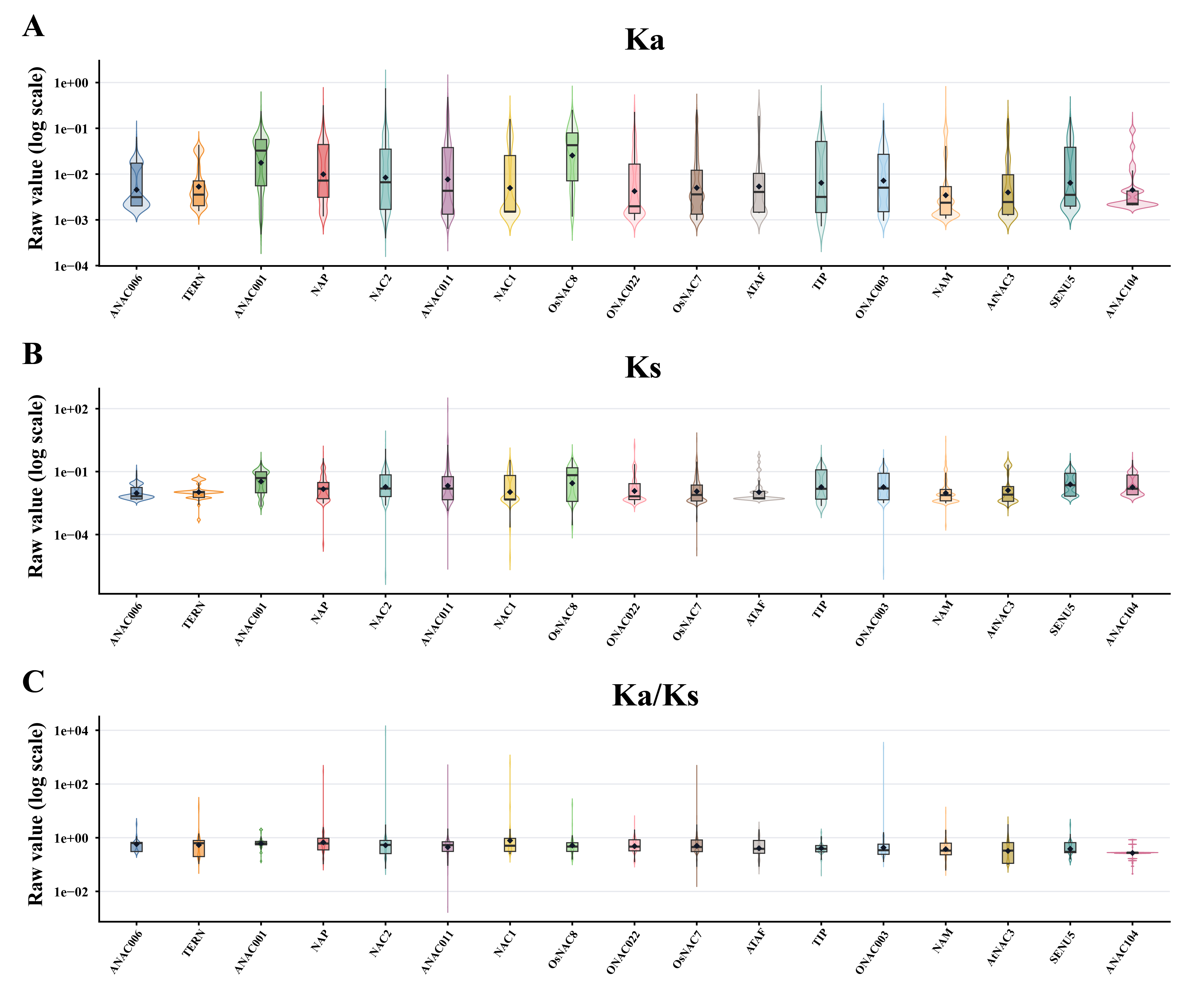

Supplement: Supplementary file 1 [file plants-15-02010-s001.zip › plants-4303159-supplementary figures/Supplementary Figures/Supplementary Figure S4.jpg]

A

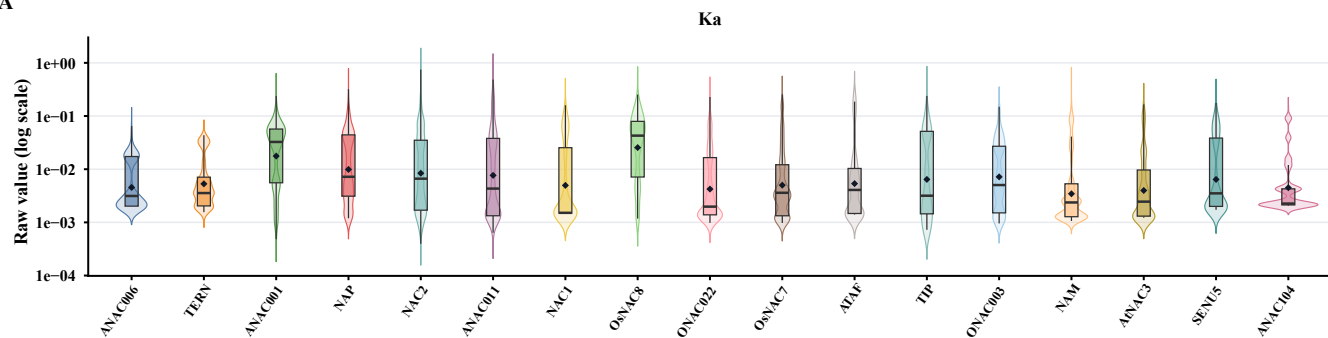

B

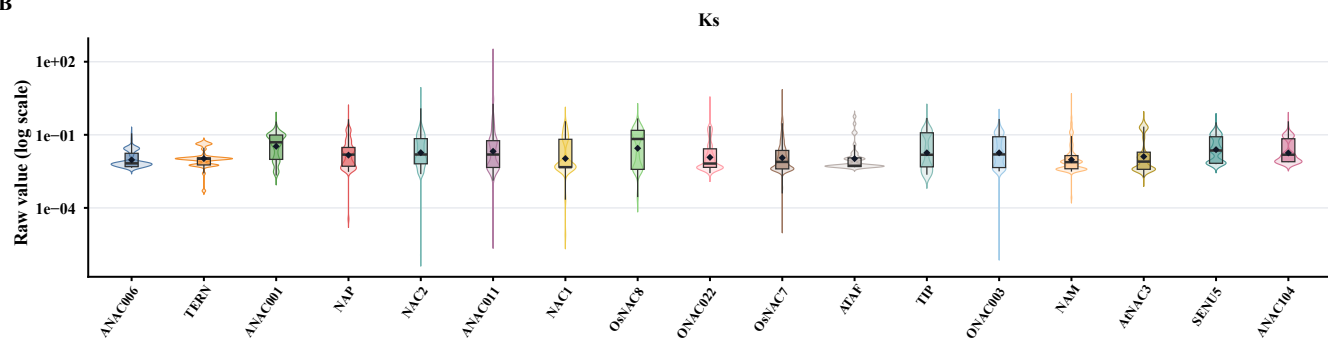

C

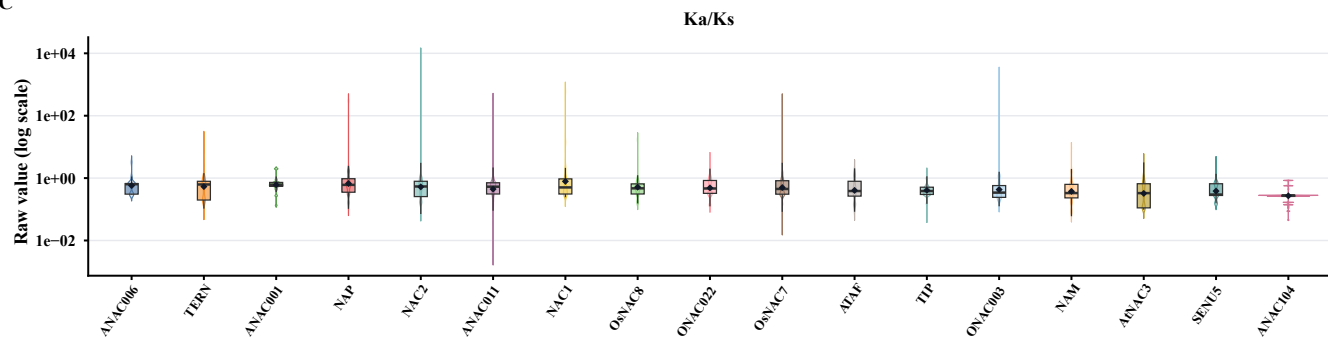

Supplement: Supplementary file 1 [file plants-15-02010-s001.zip › plants-4303159-supplementary figures/Supplementary Figures/Supplementary Figure S4.pdf]

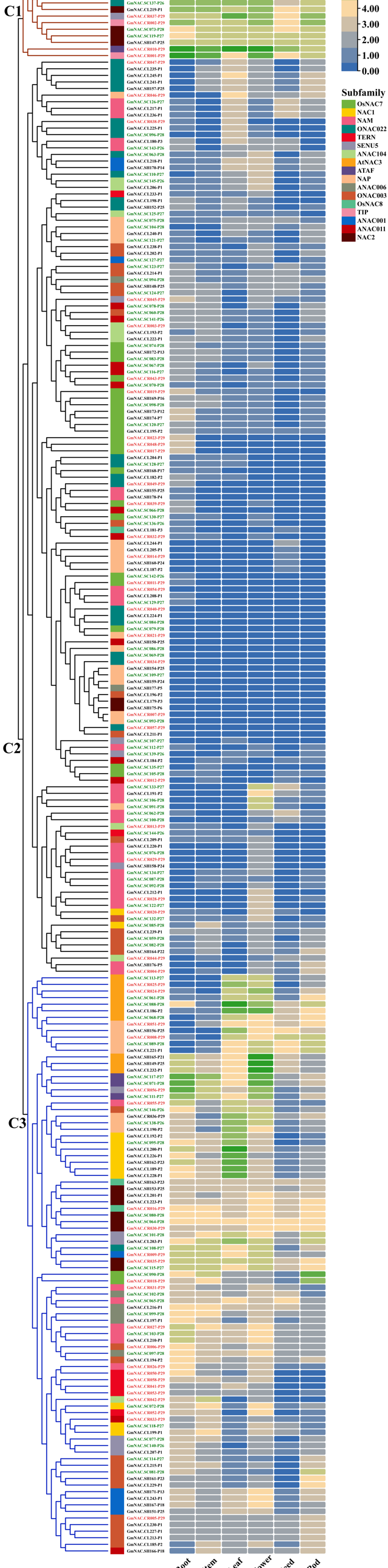

Supplement: Supplementary file 1 [file plants-15-02010-s001.zip › plants-4303159-supplementary figures/Supplementary Figures/Supplementary Figure S5.pdf]

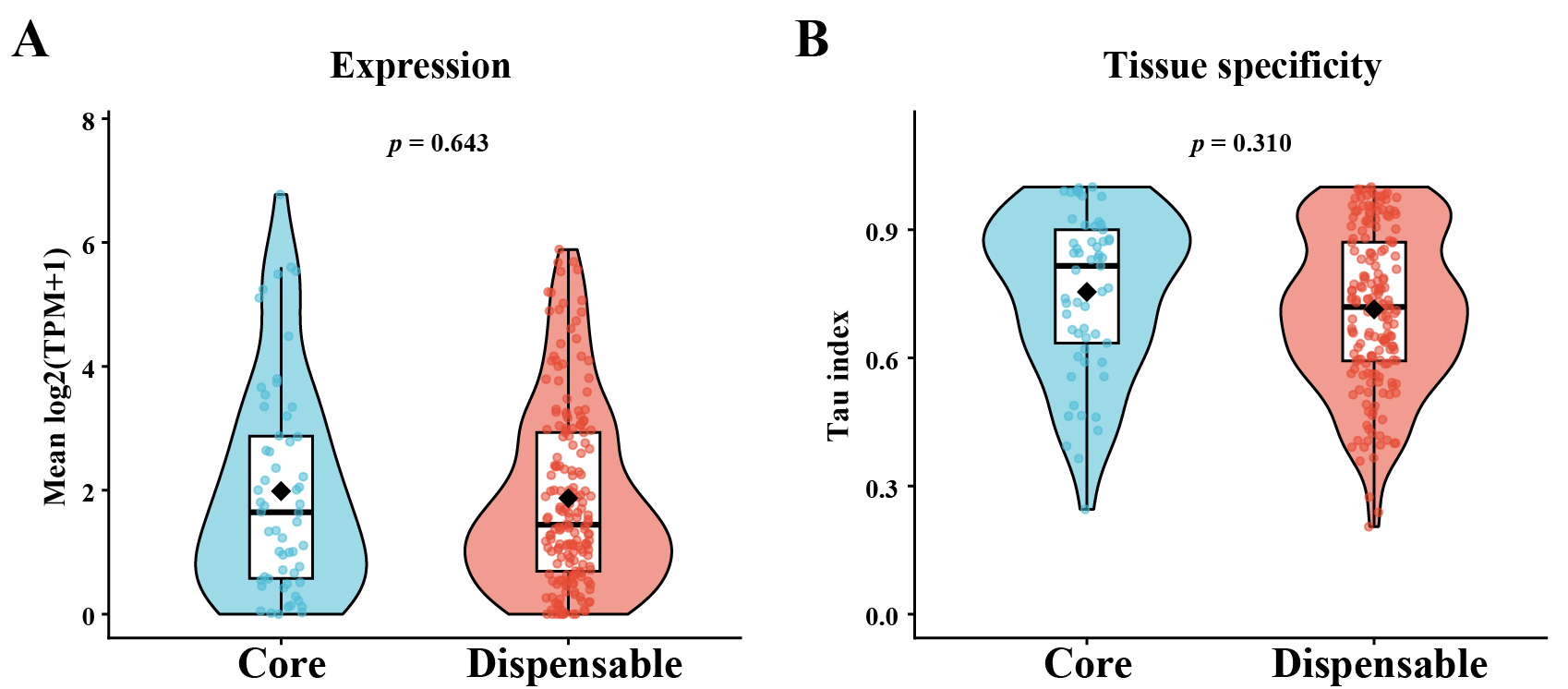

Supplement: Supplementary file 1 [file plants-15-02010-s001.zip › plants-4303159-supplementary figures/Supplementary Figures/Supplementary Figure S6.jpg]

**A****Expression**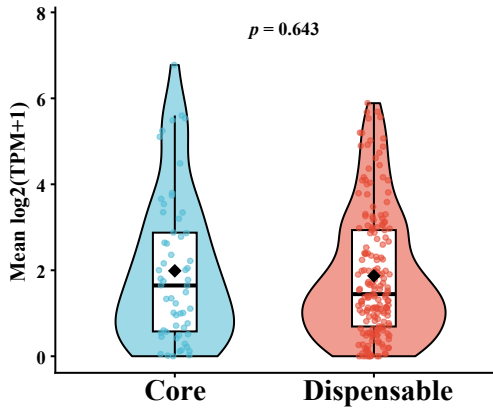**B****Tissue specificity**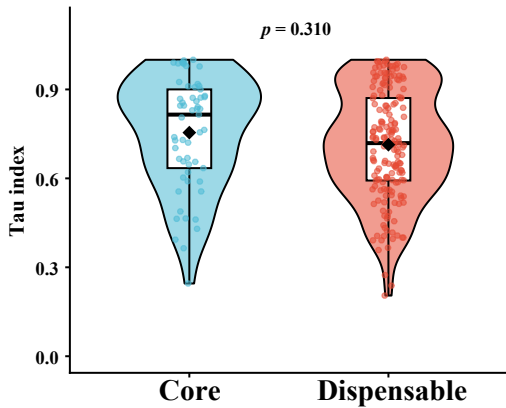

Supplement: Supplementary file 1 [file plants-15-02010-s001.zip › plants-4303159-supplementary figures/Supplementary Figures/Supplementary Figure S6.pdf]

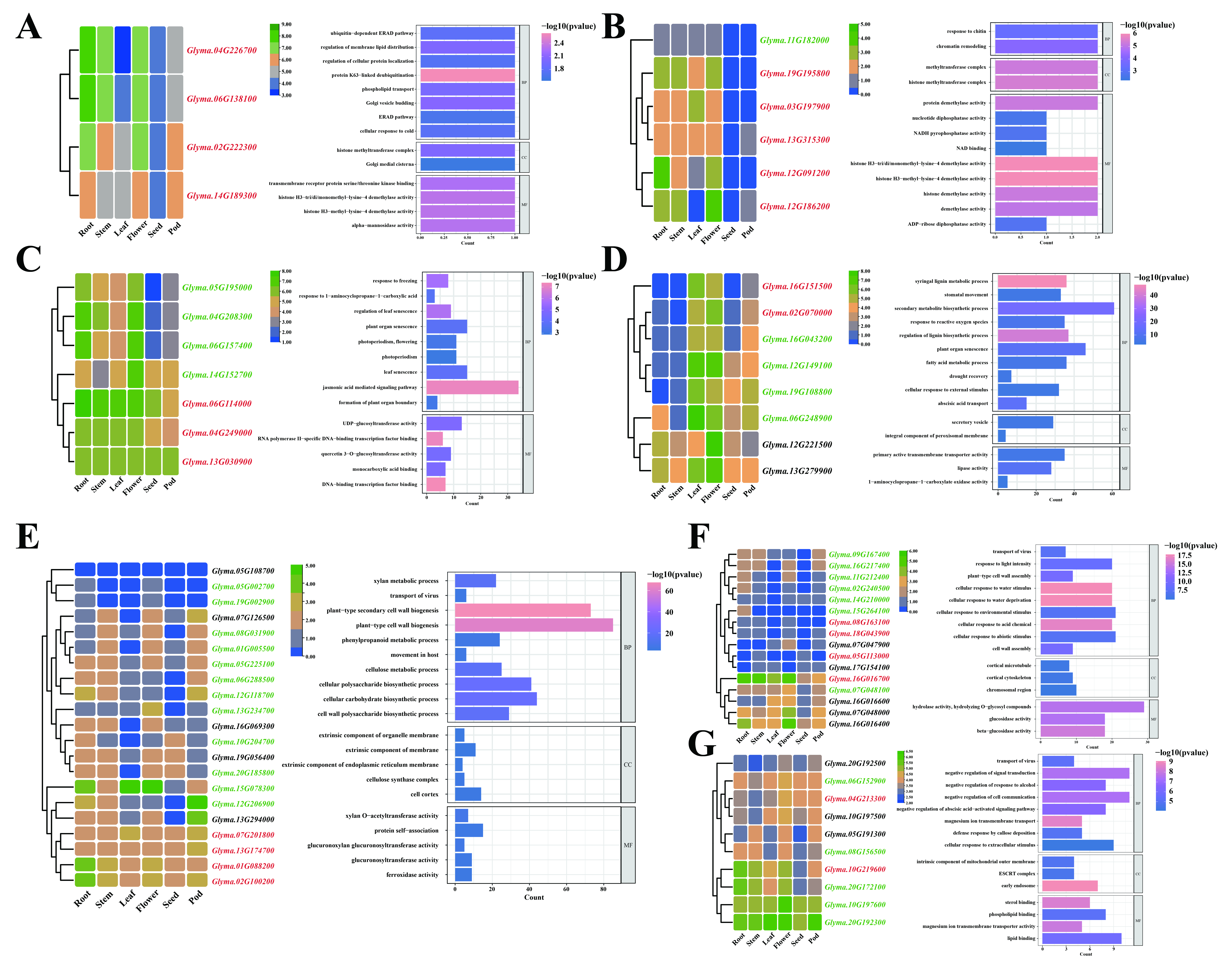

Supplement: Supplementary file 1 [file plants-15-02010-s001.zip › plants-4303159-supplementary figures/Supplementary Figures/Supplementary Figure S7.jpg]

A

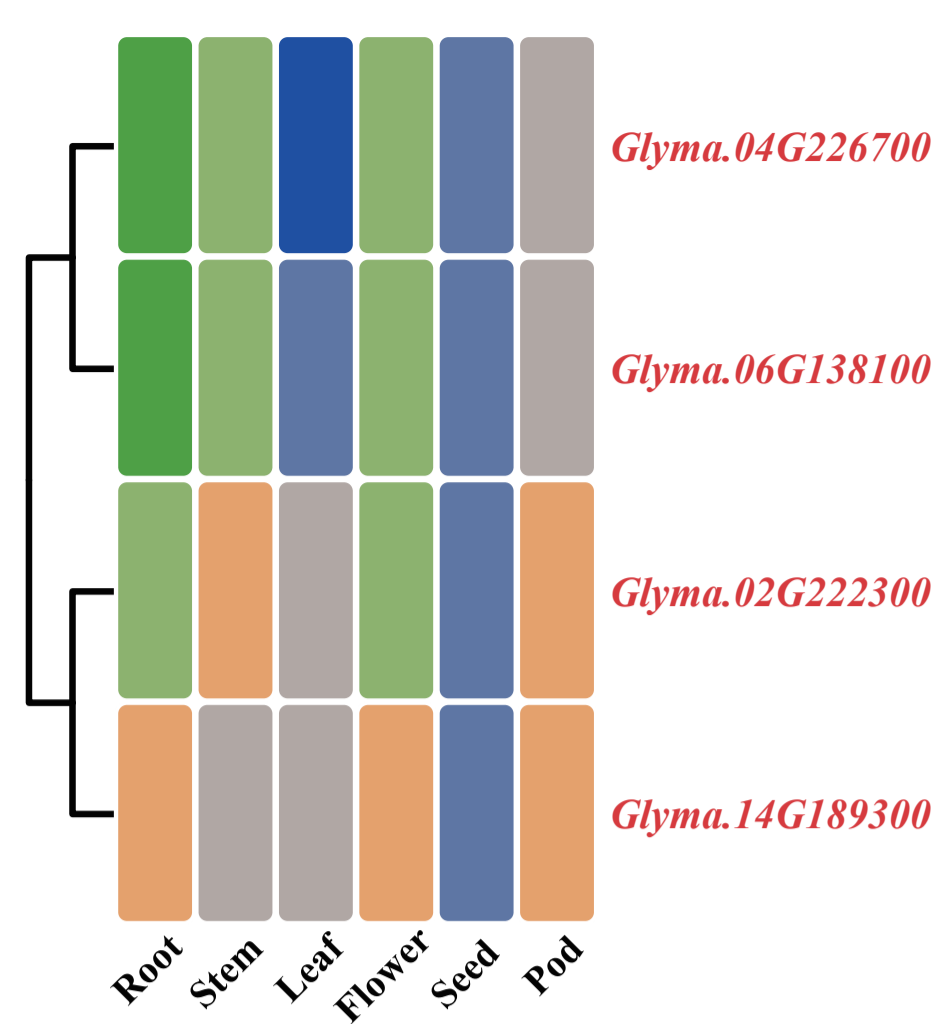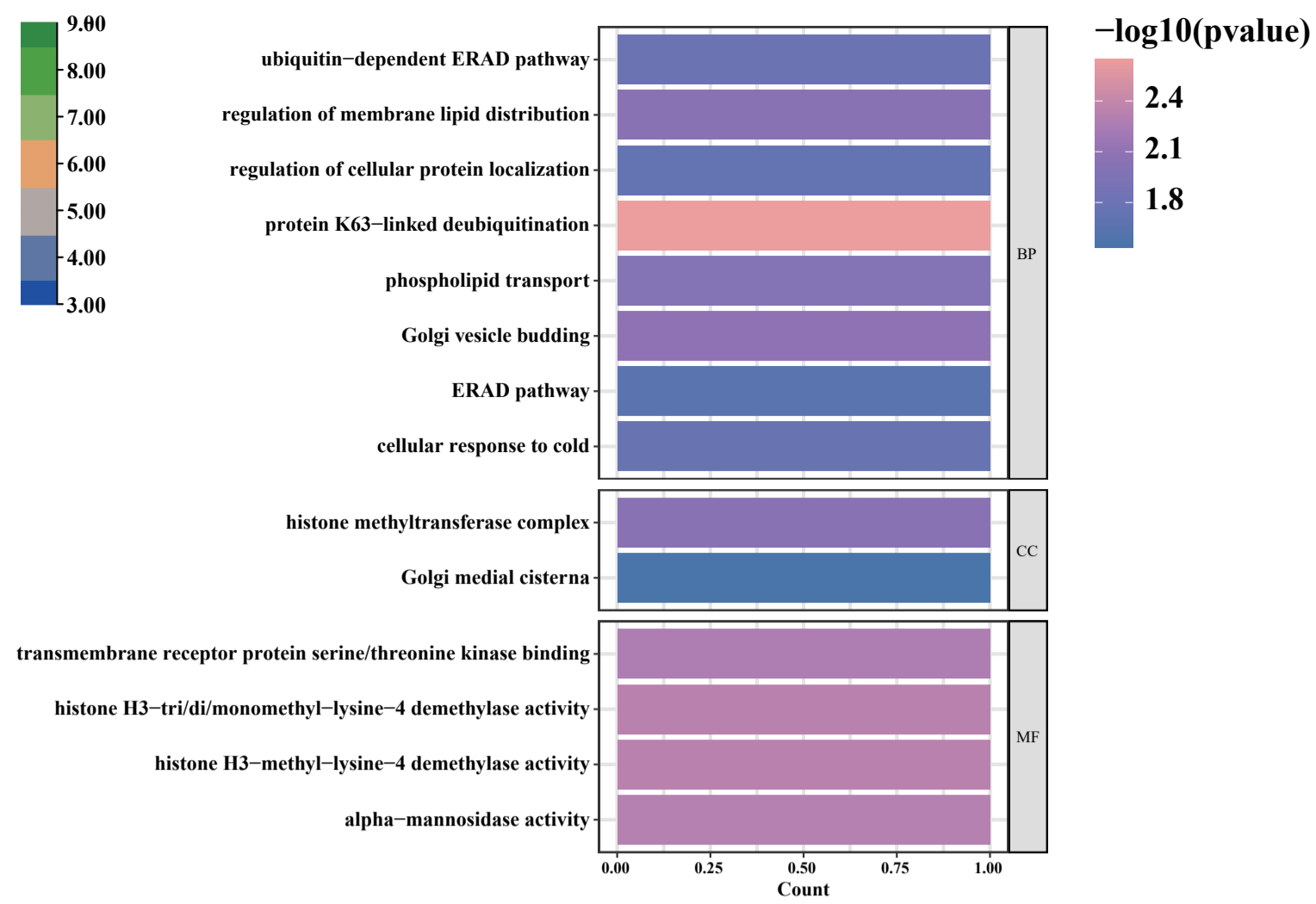

B

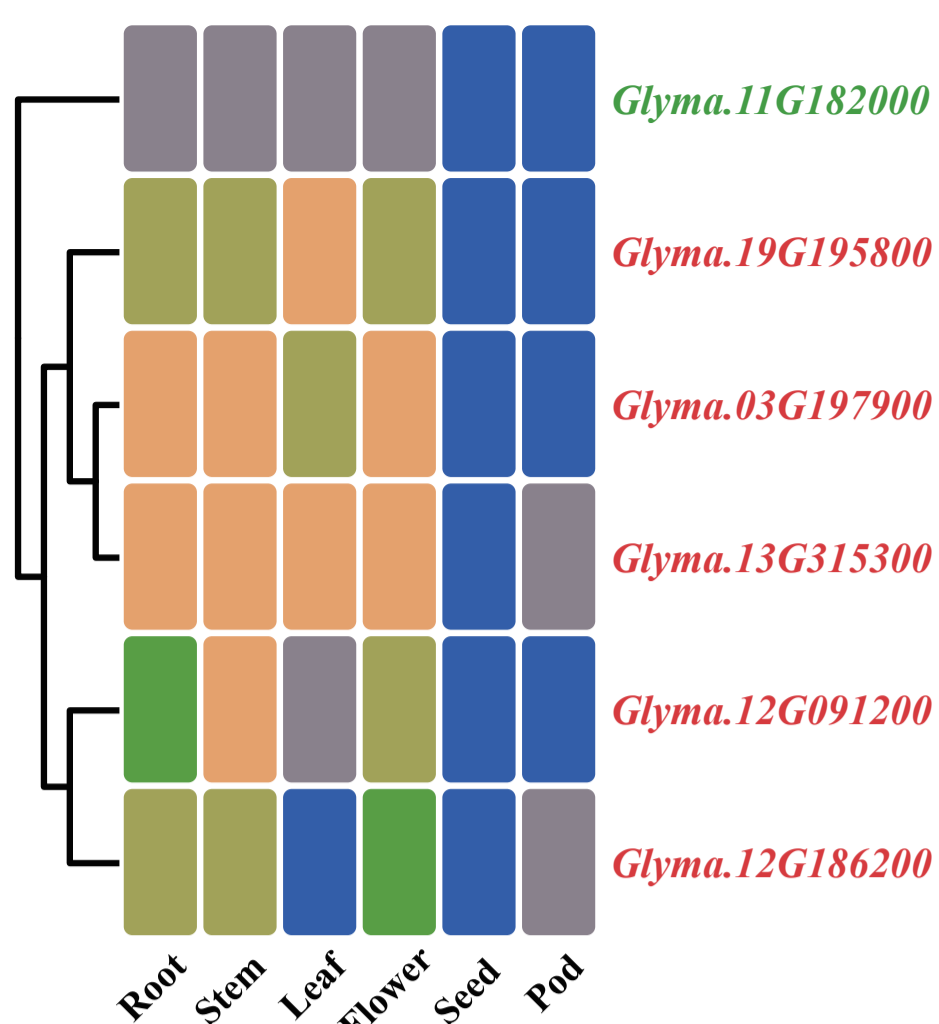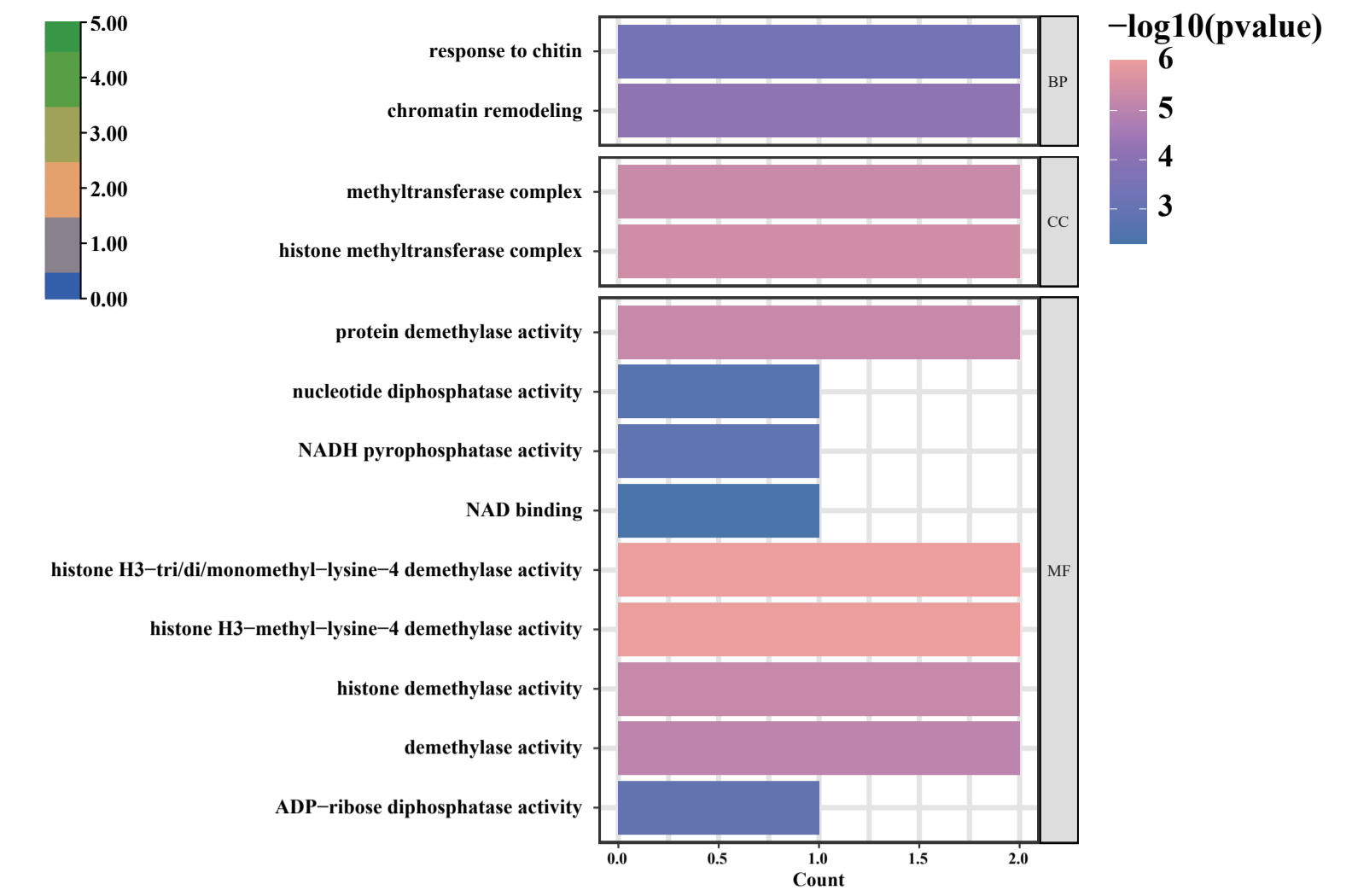

C

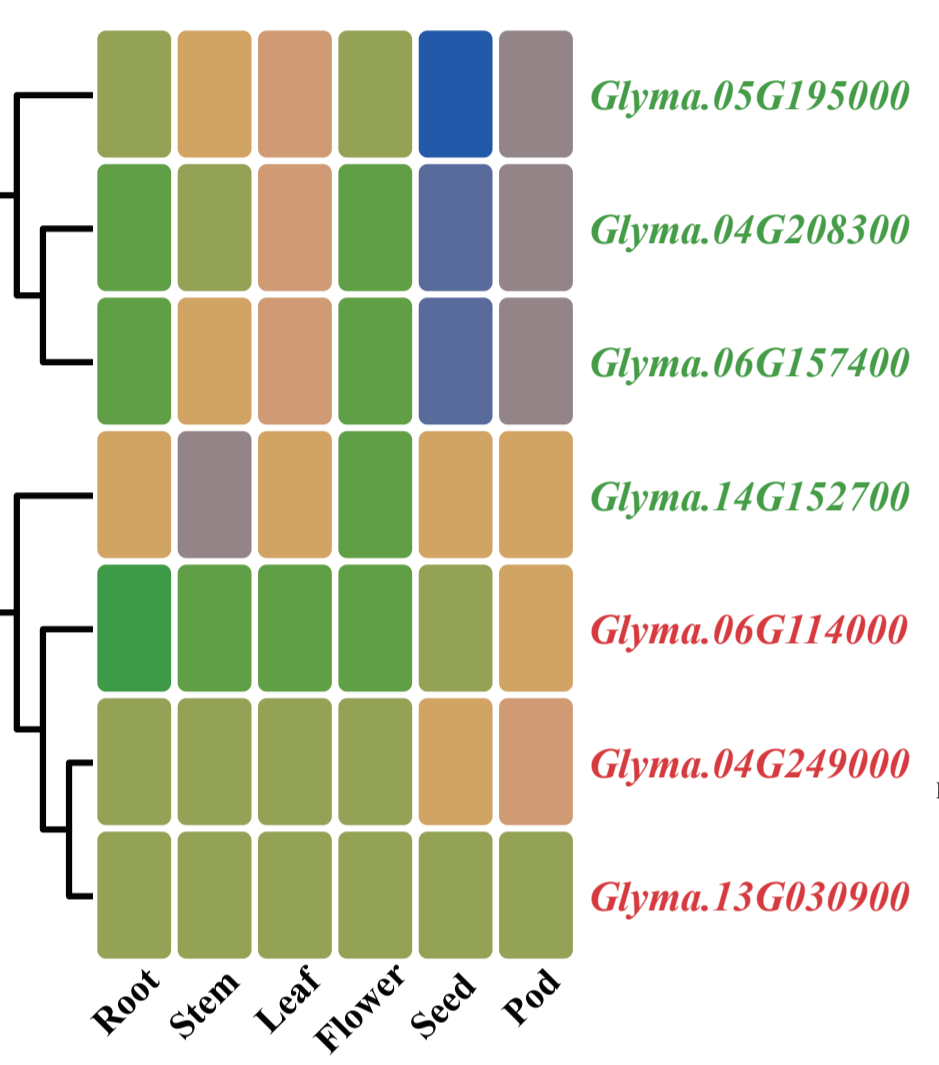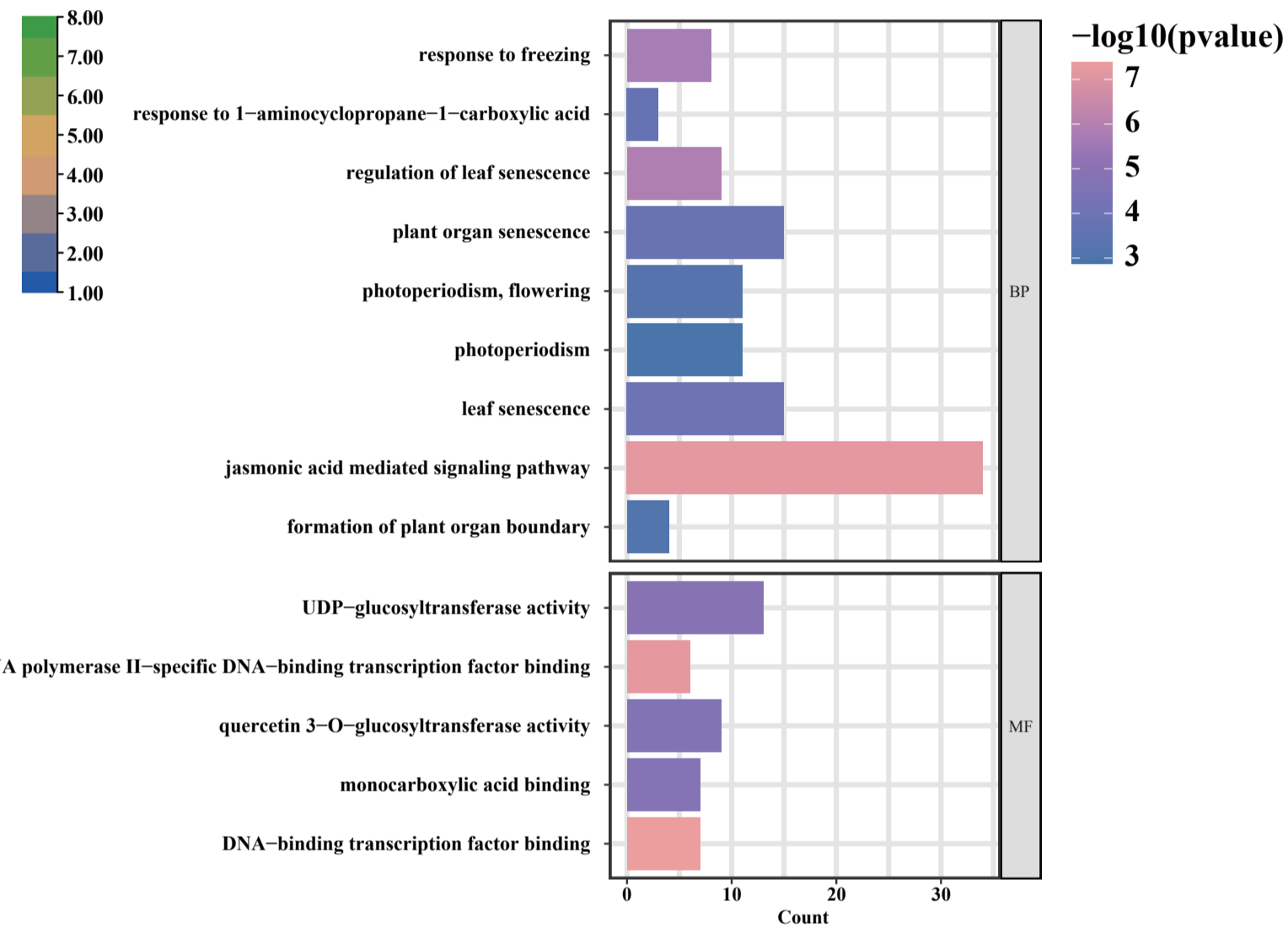

D

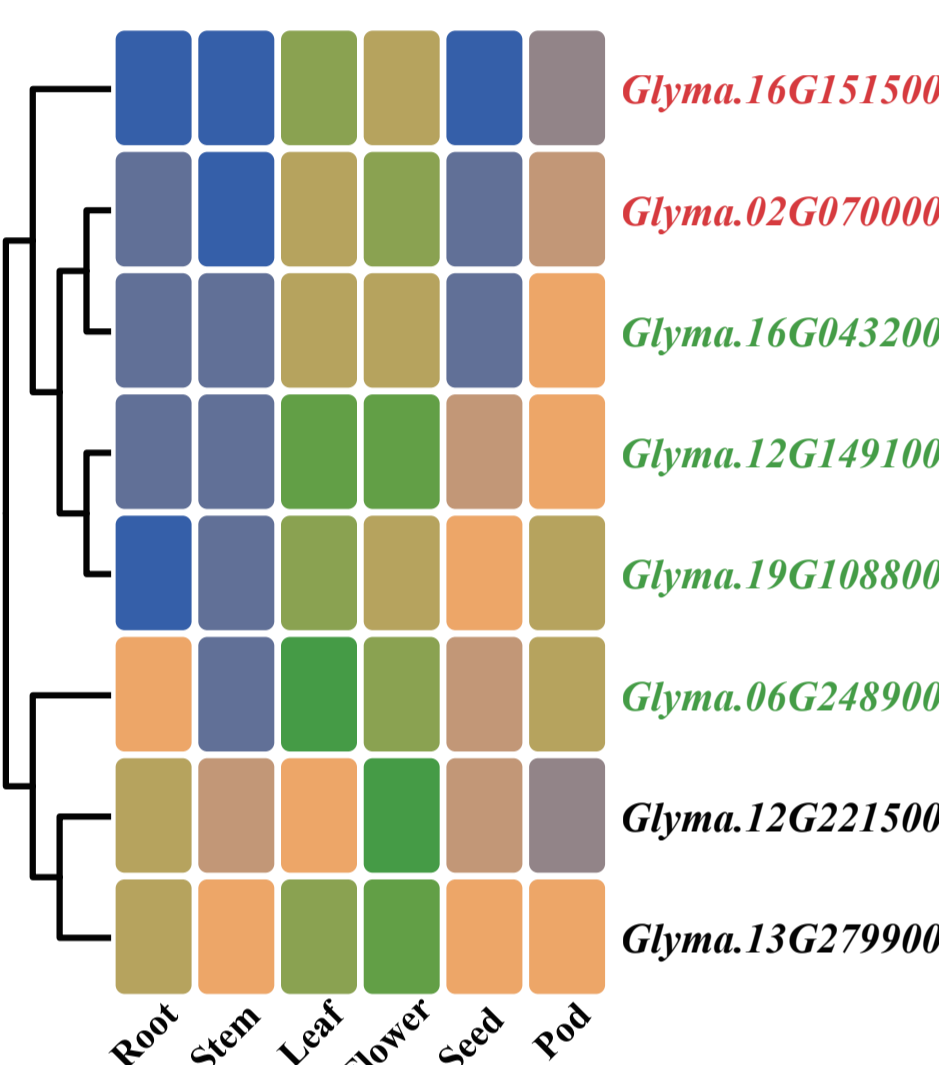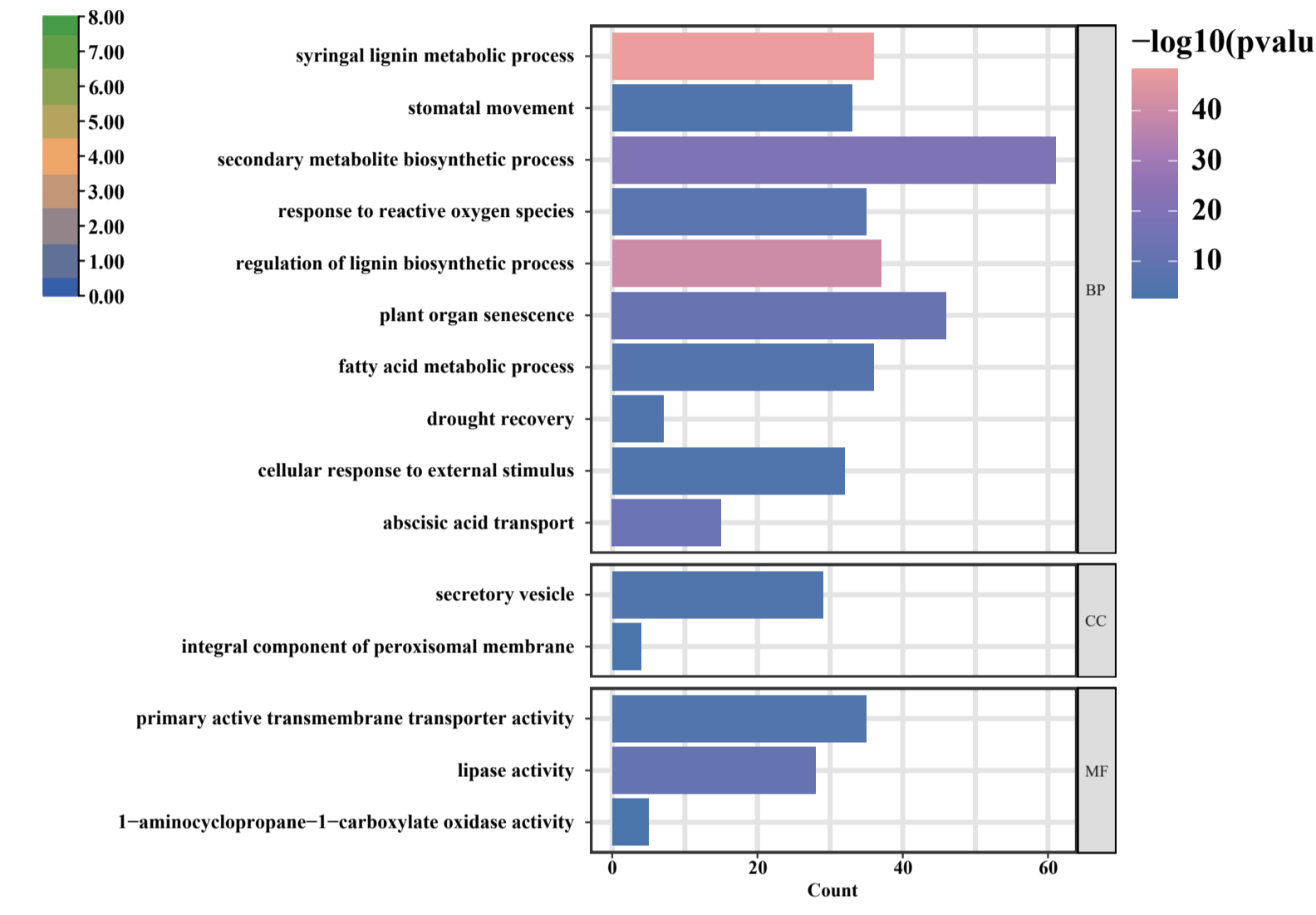

E

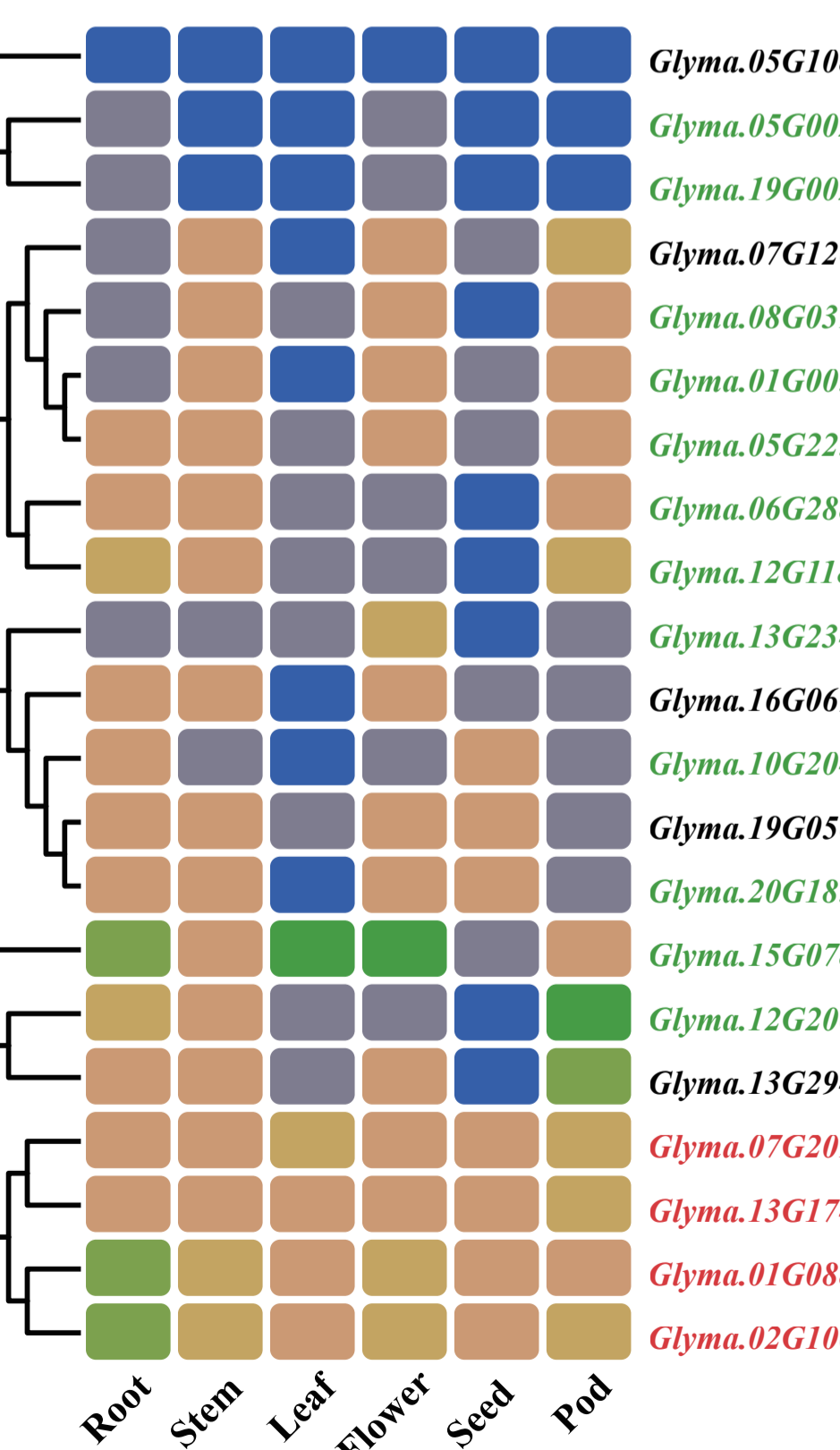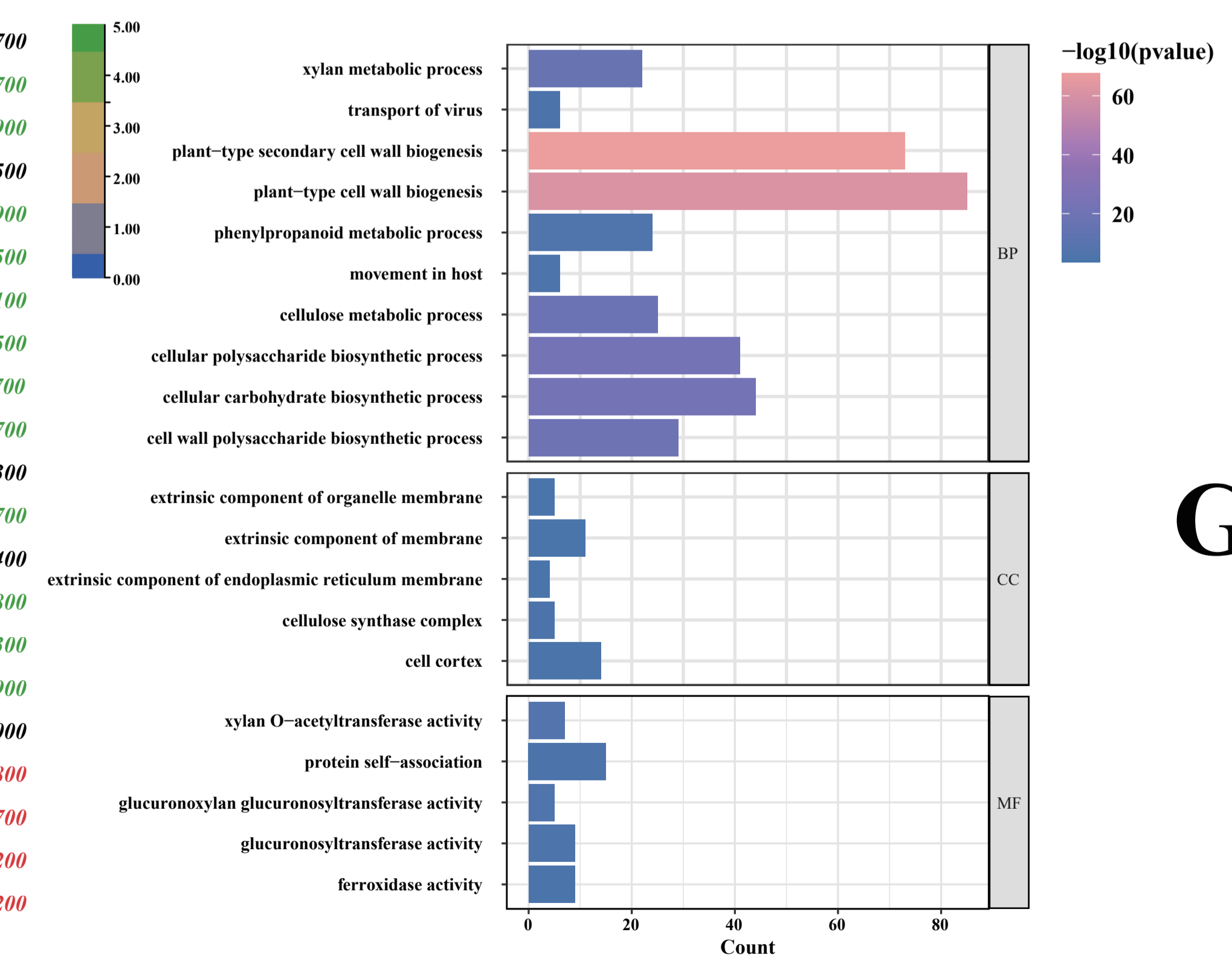

F

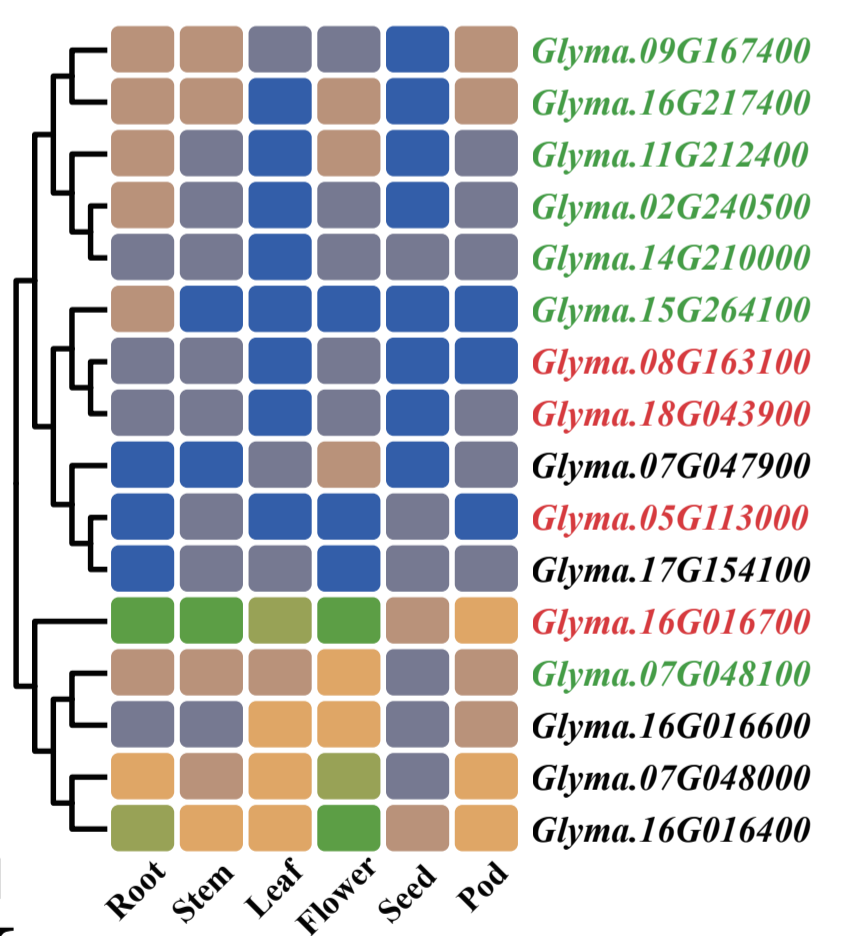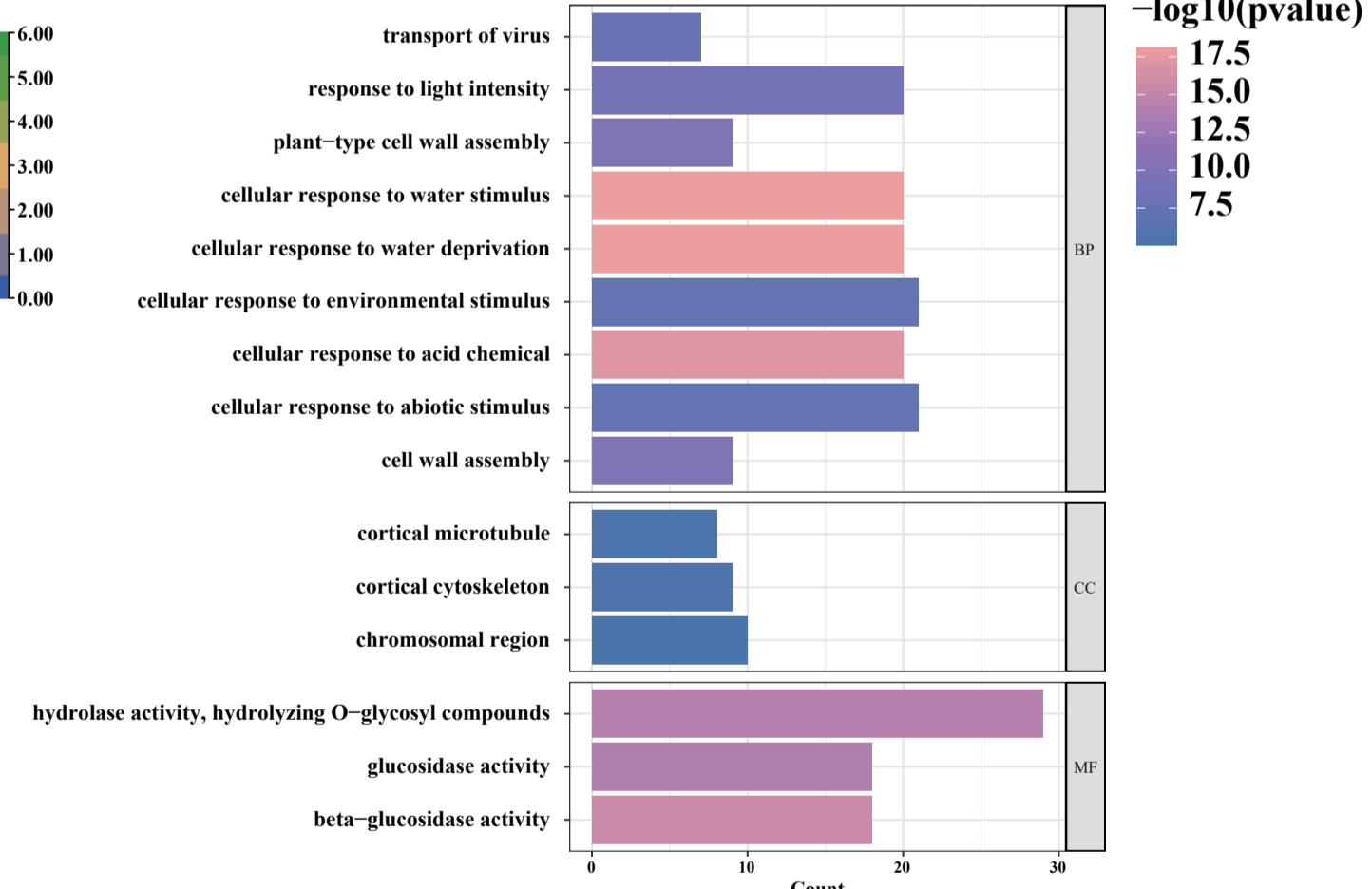

G

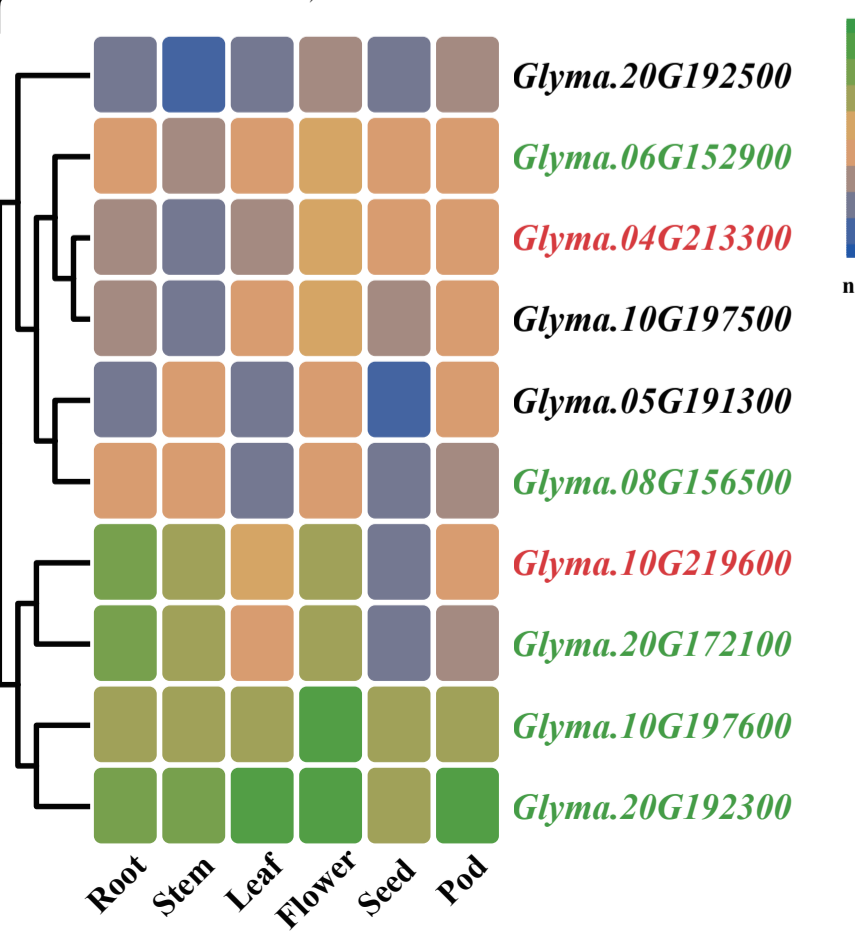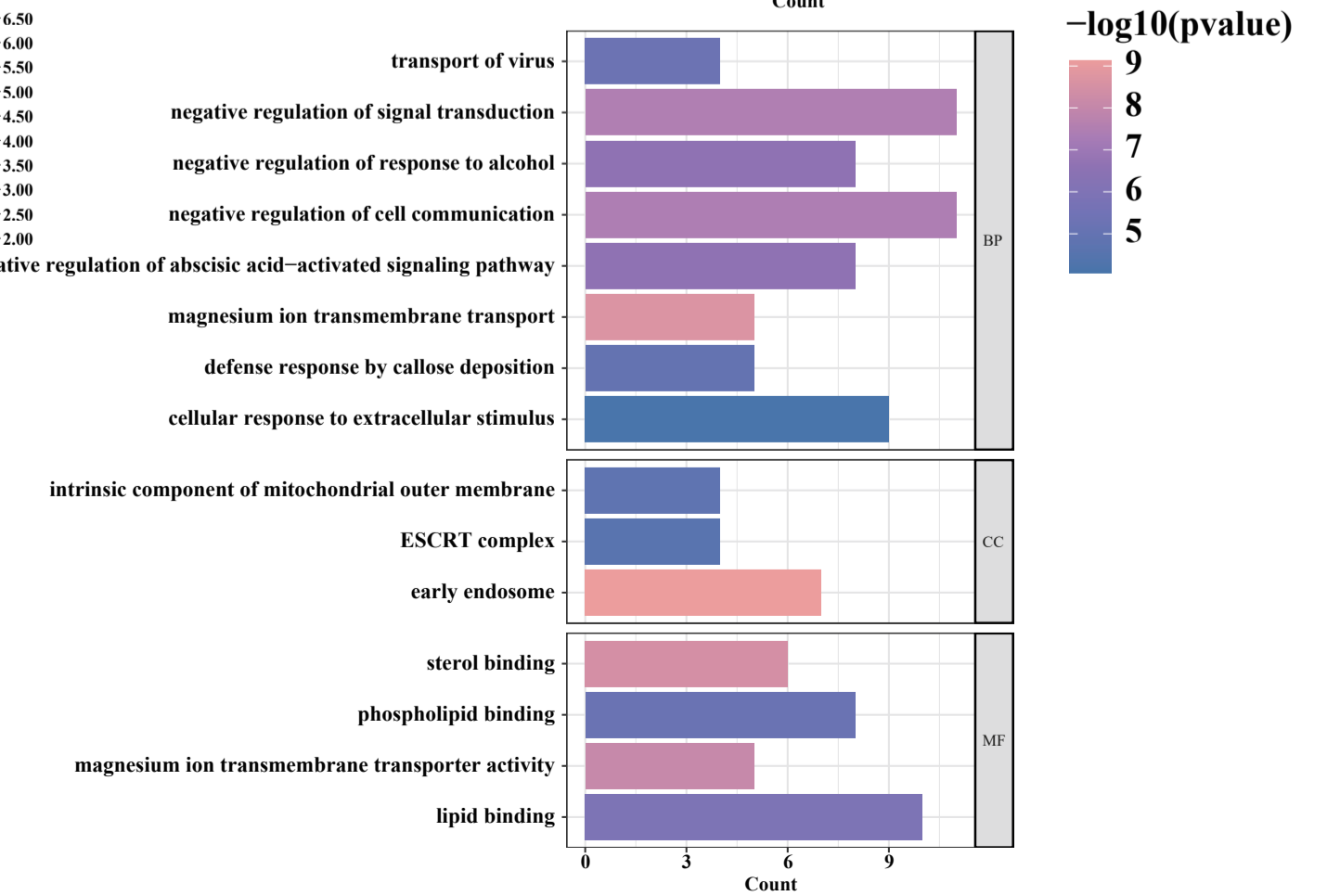

Supplement: Supplementary file 1 [file plants-15-02010-s001.zip › plants-4303159-supplementary figures/Supplementary Figures/Supplementary Figure S7.pdf]
